# Supplementary figures and images for: Does caching strategy vary with microclimate in endangered Mt. Graham red squirrels?
Source: PLoS One. 2019 Nov 12;14(11):e0224947. doi: 10.1371/journal.pone.0224947 (PMC6850554; doi:10.1371/journal.pone.0224947)

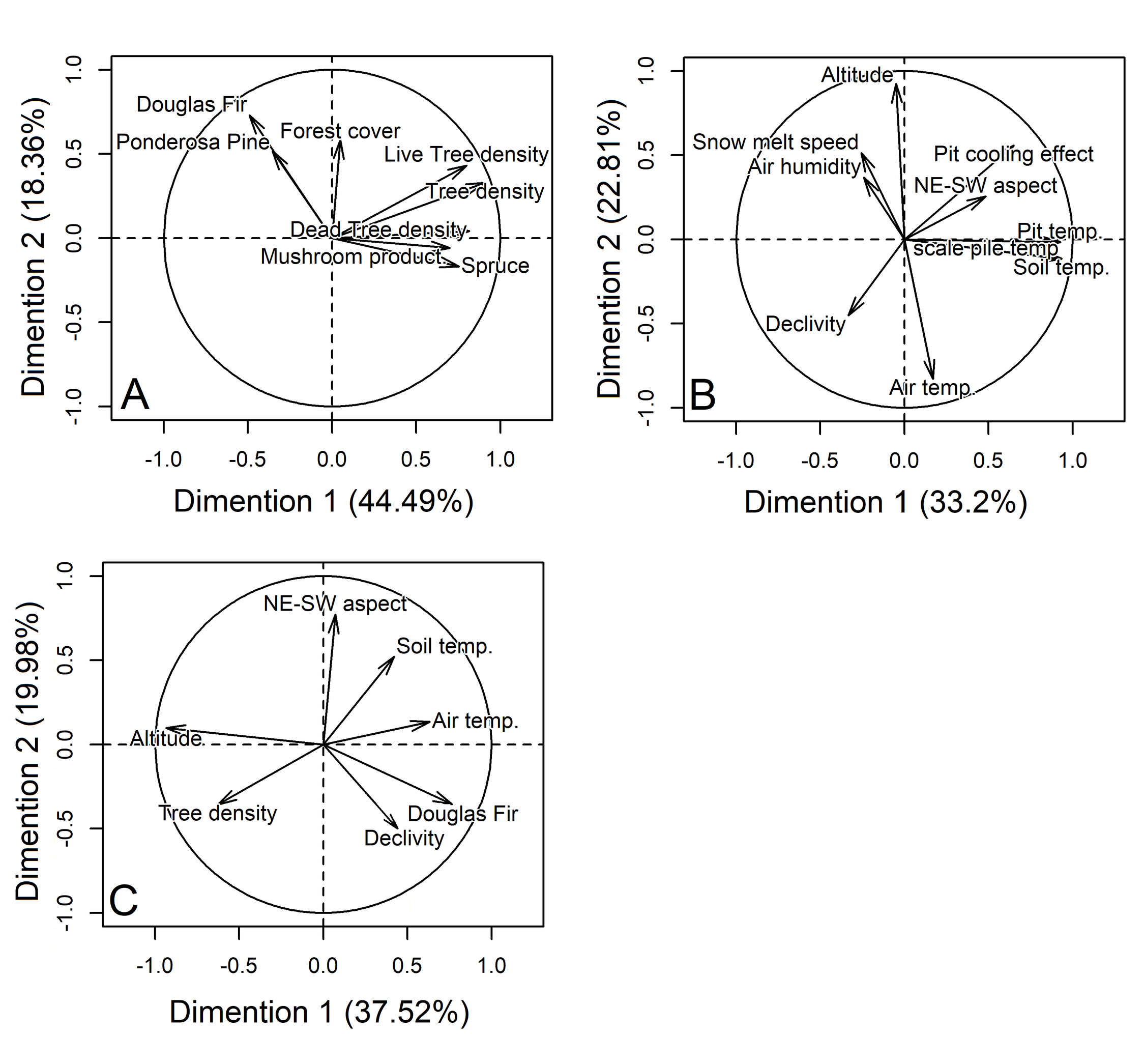

Supplement: S1 Fig — PCA, showing the multivariate variation of the biotic (a) and abiotic (b) explanatory variables. Variables with similar variation were tested for correlation by the Pearson’s correlation coefficient, when correlations were found, the most dissimilar variable was kept while the other was discarded. The resulting biotic and abiotic variables were put all together and the process was repeated (c). In the end of the process, only the four variables (tree density, soil temperature, air temperature and aspect in a Northeast-southwestern axis) remained and therefore were used in the model selection. (TIF) [file pone.0224947.s002.tif]
