# Supplementary material for: Does caching strategy vary with microclimate in endangered Mt. Graham red squirrels?
Source: PLoS One. 2019 Nov 12;14(11):e0224947. doi: 10.1371/journal.pone.0224947 (PMC6850554; doi:10.1371/journal.pone.0224947)
Supplement: Attachment — Submitted filename: PONE-D-19-16349_R1JEMrevised.pdf [file pone.0224947.s004.pdf]

PLOS ONE

Does caching strategy vary with microclimate in endangered Mt. Graham red squirrels?

--Manuscript Draft--

**Manuscript Number:**	PONE-D-19-16349R1
**Article Type:**	Research Article
**Full Title:**	Does caching strategy vary with microclimate in endangered Mt. Graham red squirrels?
**Short Title:**	Mt. Graham red squirrel cache behavior
**Corresponding Author:**	Calebe Pereira Mendes, Dh.D.<br>Universidade Estadual Paulista Júlio de Mesquita Filho<br>Rio Claro, SP BRAZIL
**Keywords:**	
**Abstract:**	Food hoarding is a common behavior used by a variety of animals to cope with periods of low food availability. At the retreating edge of species' distribution, the stressful environment and unfavourable climate conditions may impose severe costs on hoarding behavior. Since relict populations are hotspots for evolution and adaptation, and considering that food hoarding behavior has a strong evolutionary basis, we decided to evaluate the occurrence of behavioral variability in the amount of food cached by the endangered Mount Graham red squirrel (Tamiasciurus fremonti grahamensis). We tested the variation in cache size in response to microclimate, soil relief, vegetation, food availability and squirrel sex. The number of pits excavated by squirrels to cache cones was used as a proxy of cache size and was affected by mountain slope aspect and density of trees. More pits were excavated in the northeast facing slopes. The density of trees negatively affects the cache volume on southwest slopes, but not on northeast slopes. The sex of the resident squirrel also affects the number of pits in the squirrel midden, with males excavating 47% more pits than females. Males and females also presented different responses to the mountain slope aspect, with females excavating more pits on northeastern slopes than on southwestern slopes, whereas male cache size did not vary with the slope aspect. Finally, the squirrel's caching behavior did not vary in response to midden microclimate variation, a result with possible implications for the survival of the Mt Graham red squirrels, given the predicted temperature increases in the region due to climate change.
**Order of Authors:**	Calebe Pereira Mendes, Dh.D.
	John Koprowski
**Opposed Reviewers:**	
**Response to Reviewers:**	We thank the reviewers, for provide such a helpful feedback. Here are the responses to the questions.<br><br>Reviewer #1: This manuscript explores a highly fascinating behaviour 'caching variation in response to environmental and individual factors (gender)' in an endangered tree squirrel that inhabits a mountain range at the edge of the species distributional range. The latter is critical as these populations are subject to different evolutionary drivers than populations at the core of the distribution and therefore of particular interest when it comes to understanding behaviours and adaptions in a changing world.<br><br>The manuscript is exceedingly well written and a very interesting read. My only comment is that given the aim of the authors, this manuscript establishes a baseline and as a concluding thought I would encourage the research group to repeat this study in the future once climate change effects have progressed to see how this is affecting caching decisions and conclusions drawn to date. This would also show if squirrels respond to extreme temperature events. In this context a control study area at the core of the distribution not subject to extreme temperature fluctuations would be helpful for comparison.

Powered by Editorial Manager® and ProduXion Manager® from Aries Systems Corporation

Specific comments

1. Line 55: the statement about distance here lacks context. Is it really distance per se or more an increase in predation risk which makes retrieving this item for other individuals not familiar with the precise location of the valuable item more risky?

R: Indeed, the statement was unclear. We added some words to make the background clearer. But we didn’t add a complete explanation since it is provided in the cited manuscript.
Since squirrels usually cache food in the surroundings of the food source, farther places are safer in terms of conspecific pilferage. However, it takes more time and energy to travel farther before cache an item, increasing the general cost of the cache behavior by increasing the energy expended/energy retrieved (when the food is recovered) and reducing the energy cached/foraging time. This way, only more valuable food items worth the longer travel to a safer caching spot.

2. Line 57: From a European perspective it would perhaps be helpful here at first mention of this species (as opposed to Mt Graham red squirrel above) to at least once in addition to the scientific name to say the North American red or pine squirrel (Tamiasciurus hudsonicus) to avoid confusion for general readers.

R: Thank you for highlighting this issue. The words were added.

3. Line 64: should be 'depend'

R: We corrected the phrase grammar by changing the previous word (“caches” to “cache”).

4. Lines 68 and 71 - you have given Waite and Strickland 2006 here as full text when all other references are given as numbers - in your list it is at 42 and this should be changed.

R: The citations were corrected, making sure to meet the journal’s guidelines.

5. Line 111 - I wonder if these "absolute" predictions need to be modified or set into context? I assume it is a given that predictions 1 and 2 (lines 107-110) are also subject to local cone (mushroom) availability per se (your variable of density of trees in midden vicinity?

R: To avoid make unnecessarily complex predictions, we carefully created only three simple and direct predictions, each one with single predictor subject (1. Large scale temperature, 2. microclimate, 3. food availability). Yes, we indeed expected these predictions to interfere with each other, and this is the reason why we make sure to use non-excluding predictions (the three can be truth and have summed effects). We even tested these interactions in the methods to some degree, always being careful to choose a less demanding statistic approach due to the limited sample size.

6. Line 196 'varied' should be 'vary'

R: The word was changed.

7. Lines 196 to 199 - as aspect is a critical factor in your later findings, did you include aspect as a variable here - did/would the sample of 73 trees allow this? One underlying assumption in your study is that all trees within midden vicinity are equal in terms of food availability and their density serves as a proxy. One might in principle expect aspect to matter in terms of cone production and this should be checked.

Powered by Editorial Manager® and ProduXion Manager® from Aries Systems Corporation

R: Thank you for highlight this issue. We added the information about the possible correlation between cone production and mountain aspect. The cones production was also not correlated with aspect.

Reviewer #2: This paper describes an important evolutionary adaptation: behavioural variability in the amount of food cached by the endangered Mount Graham red squirrel (Tamiasciurus fremonti grahamensis). Authors tested the variation in cache size in response to microclimate variation, based on the following parameters: relief, vegetation (tree stand density), food availability and squirrel sex. Based on assumptions and predictions obtained from previous studies scientists expected that squirrels should locate bigger caches in areas:
1. Where the conditions to cone preservation are better and spoilage is expected to be reduced (colder areas).
2. With colder microclimates: in higher elevations and in slopes facing the northeast.
3. With higher cone availability.
To verify these hypothesis authors selected 40 middens occupied by squirrels distributed along different altitudes, relief, and mountain aspect. They visited each selected midden six times between the fall and winter of 2015.
Results of the study show, that much more pits were excavated in the northeast facing slopes. Surprisingly, the density of trees negatively affects the cache volume on southwest slopes only. The sex of the squirrel also affects the number of pits in the squirrel midden, males excavating more pits than females. Additionally, both sexes presented different responses to the mountain slope aspect (females excavating more pits on northeastern slopes, whereas male cache size did not vary with the slope aspect). Contrary to authors' predictions the squirrel's caching behaviour did not vary in response to midden microclimate variation.
However, although both the idea and conducted study are very interesting and worth publication, there are some aspects which require further explanation.
First of all, in my opinion (and as the authors mentioned in the discussion), the sample size may be not big enough for such multifactorial analysis. The second question is a type of distribution of studied middens – does it cover all habitat variability?

R: Indeed, due to the unexpected high mortality of squirrels during the winter, we worked always with the limitations of a small sample size in mind, choosing the less demanding statistical approaches, such reducing the number of variables before compare the models and never creating models with more than independent 2 variables. Despite our efforts, there is a limit to what is possible to do, and this is the reason why, in good faith, we added the warning in the discursion section (as mentioned by the reviewer).
About the distribution of the study middens, since the squirrel distribution is very limited, the sampling did cover most the habitat variability. We added a phrase in the manuscript about the subject. The middens coordinates are also provided in the supplementary material.

For me, the relationship between altitude, microclimate and the number of pits in the squirrel's midden is the most important and missing information which may help us understand behavioural adaptation to the climate change. Authors should expect differences in caching squirrels' behaviour in regard to differences in elevation gradient. In my opinion this aspect should be deeply analysed and discussed. Maybe squirrels territories were located at the same elevation – it should be disclosed.

R: The altitude of the middens varied between 2770 and 3270 meters (as mentioned in the "study area" subsection), which is almost the entire range of altitude of the population. However, Altitude was not "deeply analyzed", because it was not a good predictor of squirrel cache size and was dropped in the variable reduction part of the analysis.
We added an entire paragraph to better explain the issue in the manuscript.

I am also wondering why authors analyzed differences between squirrels' sexes? Did they expect any sex-related differences in catching behaviour and if so, there should be an explanation.

It would be good to introduce potential readers for studied species biology and describe briefly the subject of the study, including sexual differences, body mass and social system.

R: We added more details about how the squirrel sexual differences and why it is expected to affect the animal dependence on cached food.

When you try to consider what causes could lead to the obtained results – the lack of behavioural reaction on micro-climate variability – the good way is to analyze the used methodology. It is possible that daily air temperature, which was measured in the meteorological station differs slightly from the daily air temperature in other places, like middens. The same could be applied to soil temperature monitored by data-logger between one (the same) place and other places (e.g. middens).
Other explanation which the author discussed in the manuscript is that the most important for catching behaviour was the maximum, not the average air temperature.

R: Indeed, we agree with the reviewer, and it was the used approach. But we did not discuss about possible differences between the measurements between the meteorological station and the middens, since this would result in constant error along the middens, and therefore, it would be automatically corrected by the used methods. As the reviewer mentioned, we preferred to discuss about possible extreme events (such as temperature peaks), which would require a very different sampling method, and therefore, are beyond the original design of the present study.

Abstract
P2 L22 - …" in response to climate"… - should be "in response microclimate"

R: the phrase was changed to "in response to microclimate"

Materials and Methods
P6 L131 – 134
"We visited each midden 6 times between the fall and winter of 2015 to measure variables of weather, relief, plant structure and estimate the volume of cones stored by the resident squirrels (response variable). During the visits, we also record the sex of the
resident squirrels."

Could you please explain how did you record the sex of the resident squirrels?
It's unclear – Did you measure all of these variables during each of the six visits? If not please add the information when did you measure each variable. As I understood the temperature in all middens was measured on the same day.
How did you choose three pits from one midden for temperature monitoring?
How accurate was your equipment for the weather variables (handheld weather meter Kestrel 3000) and a digital soil thermometer (HANNA HI45-30), it's possible that low accuracy can mask differences in temperature and humidity.

R: The sex of the resident squirrels was recorded by direct observation (using a binocular). It is a very easy and reliable method for sexing squirrels since they are usually up in the branches. We added the information in the manuscript.
We also add the information about when each variable was collected.
We didn't think the equipment precision was a problem, since it is a very reliable equipment. The technical specifications can be accessed in the respective equipment websites (±0,27°C for the HANNA HI45-30 and ± 0.04C for the Kestrel 3000).

Discussion
In this part of the manuscript, there is no explanation of why sex should generate differences in squirrel's catching behaviour.

R: The information was added in the methods section, as the reason of why we recorded squirrel sex.

	Additionally, the authors’ hypothesis about different levels in predation risk between places with high and low tree steam density as an explanation in differences in squirrels’ catching behaviour seems to be unconvincing. First of all, it is hard to believe that predation risk can vary between different mountain slope aspects.<br><br>R: We thank the reviewer to point that the argument was not clear. We make some changes to make it clearer. Indeed, the argument is not intended to explain the different responses of males and females to the mountain aspect, but only in relation to tree density.
**Additional Information:**	
**Question**	**Response**
**Financial Disclosure**<br><br>Enter a financial disclosure statement that describes the sources of funding for the work included in this submission. Review the submission guidelines for detailed requirements. View published research articles from *PLOS ONE* for specific examples.<br><br>This statement is required for submission and **will appear in the published article** if the submission is accepted. Please make sure it is accurate.<br><br>**Unfunded studies**<br>Enter: *The author(s) received no specific funding for this work.*<br><br>**Funded studies**<br>Enter a statement with the following details:<br>• Initials of the authors who received each award<br>• Grant numbers awarded to each author<br>• The full name of each funder<br>• URL of each funder website<br>• Did the sponsors or funders play any role in the study design, data collection and analysis, decision to publish, or preparation of the manuscript?<br>• **NO** - Include this sentence at the end of your statement: *The funders had no role in study design, data collection and analysis, decision to publish, or preparation of the manuscript.*<br>• **YES** - Specify the role(s) played.<br><br>* typeset	CPM received a studentship from CAPEs (Coordenação de Aperfeiçoamento de Pessoal de Nível Superior - https://www.capes.gov.br), process number: BEX 0659/15-0. The funders had no role in study design, data collection and analysis, decision to publish, or preparation of the manuscript.
**Competing Interests**	The authors have declared that no competing interests exist.

Use the instructions below to enter a competing interest statement for this submission. On behalf of all authors, disclose any competing interests that could be perceived to bias this work—acknowledging all financial support and any other relevant financial or non-financial competing interests.<br><br>This statement **will appear in the published article** if the submission is accepted. Please make sure it is accurate. View published research articles from *PLOS ONE* for specific examples.<br><br>**NO authors have competing interests**<br><br>Enter: *The authors have declared that no competing interests exist.*<br><br>**Authors with competing interests**<br><br>Enter competing interest details beginning with this statement:<br><br>*I have read the journal's policy and the authors of this manuscript have the following competing interests: [insert competing interests here]*<br><br>* typeset	
**Ethics Statement**<br><br>Enter an ethics statement for this submission. This statement is required if the study involved:<br><br>• Human participants<br>• Human specimens or tissue<br>• Vertebrate animals or cephalopods<br>• Vertebrate embryos or tissues<br>• Field research<br><br>Write "N/A" if the submission does not require an ethics statement.<br><br>General guidance is provided below. Consult the submission guidelines for detailed instructions. **Make sure that all**	The study was conducted under permits from the Arizona Game and Fish Department (permit number SP696903), the US Fish and Wildlife Endangered Species (permit number TE041875), and the University of Arizona's Institutional Animal Care and Use Committee (permit number #14-504).

Powered by Editorial Manager® and ProduXion Manager® from Aries Systems Corporation

**information entered here is included in the Methods section of the manuscript.**<br><br>**Format for specific study types**<br><br>**Human Subject Research (involving human participants and/or tissue)**<br>• Give the name of the institutional review board or ethics committee that approved the study<br>• Include the approval number and/or a statement indicating approval of this research<br>• Indicate the form of consent obtained (written/oral) or the reason that consent was not obtained (e.g. the data were analyzed anonymously)<br><br>**Animal Research (involving vertebrate animals, embryos or tissues)**<br>• Provide the name of the Institutional Animal Care and Use Committee (IACUC) or other relevant ethics board that reviewed the study protocol, and indicate whether they approved this research or granted a formal waiver of ethical approval<br>• Include an approval number if one was obtained<br>• If the study involved *non-human primates*, add *additional details* about animal welfare and steps taken to ameliorate suffering<br>• If anesthesia, euthanasia, or any kind of animal sacrifice is part of the study, include briefly which substances and/or methods were applied<br><br>**Field Research**<br><br>Include the following details if this study involves the collection of plant, animal, or other materials from a natural setting:<br>• Field permit number<br>• Name of the institution or relevant body that granted permission	
**Data Availability**<br><br>Authors are required to make all data underlying the findings described fully available, without restriction, and from the time of publication. PLOS allows rare exceptions to address legal and ethical	Yes - all data are fully available without restriction

concerns. See the PLOS Data Policy and FAQ for detailed information.<br><br>A Data Availability Statement describing where the data can be found is required at submission. Your answers to this question constitute the Data Availability Statement and **will be published in the article**, if accepted.<br><br>**Important:** Stating 'data available on request from the author' is not sufficient. If your data are only available upon request, select 'No' for the first question and explain your exceptional situation in the text box.<br><br>Do the authors confirm that all data underlying the findings described in their manuscript are fully available without restriction?	
**Describe where the data may be found in full sentences. If you are copying our sample text, replace any instances of XXX with the appropriate details.**<br><br>• If the data are **held or will be held in a public repository**, include URLs, accession numbers or DOIs. If this information will only be available after acceptance, indicate this by ticking the box below. For example: *All XXX files are available from the XXX database (accession number(s) XXX, XXX.*).<br>• If the data are all contained **within the manuscript and/or Supporting Information files**, enter the following: *All relevant data are within the manuscript and its Supporting Information files.*<br>• If neither of these applies but you are able to provide **details of access elsewhere**, with or without limitations, please do so. For example:<br><br>*Data cannot be shared publicly because of [XXX]. Data are available from the XXX Institutional Data Access / Ethics Committee (contact via XXX) for researchers who meet the criteria for access to confidential data.*	All relevant data are within the manuscript and its Supporting Information files.

The data underlying the results presented in the study are available from (include the name of the third party and contact information or URL).<br>• This text is appropriate if the data are owned by a third party and authors do not have permission to share the data.<br><br>* typeset	
Additional data availability information:	Tick here if the URLs/accession numbers/DOIs will be available only after acceptance of the manuscript for publication so that we can ensure their inclusion before publication.

Powered by Editorial Manager® and ProduXion Manager® from Aries Systems Corporation

unesp

UNIVERSIDADE ESTADUAL PAULISTA
“JÚLIO DE MESQUITA FILHO”
Instituto de Biociências – Campus de Rio Claro

Departamento de Ecologia

September 2nd, 2019

PLOS ONE

Editor-in-Chief Joerg Heber

Dear Dr. Heber,

Please find attached the revised version of our manuscript (PONE-D-19-16349 - “*Does caching strategy vary with microclimate in endangered Mt. Graham red squirrels?*”). We made all the changes required by the reviewers and responded all the questions. For each question, we added more information in the manuscript to make the issue clearer. The citations were also corrected to meet the PLOS ONE's style requirements.

Thank you for your consideration, and if you need further information, please do not hesitate to contact us.

Yours sincerely,

Calebe Mendes

Corresponding author: Calebe Mendes (calebepm3@hotmail.com)

Laboratório de Biologia da Cobservação (LABIC), Instituto de Biociências, Universidade Estadual Paulista “Júlio de Mesquita Filho” (UNESP - RC), 13506-900, Rio Claro, SP., Brasil.

Manuscript
Click here to access/download;Manuscript;Manuscript.docx

Does caching strategy vary with microclimate in endangered Mt. Graham red squirrels?

Calebe Pereira Mendes[1]*, and John Koprowski[2]

[1] *Laboratório de Biologia da Conservação, Instituto de Biociências, Universidade Estadual Paulista "Júlio Mesquita Filho" (UNESP), Rio Claro, São Paulo*, Brazil.

[2] *School of Natural Resources and the Environment, University of Arizona, Tucson, Arizona, United States of America.*

* Corresponding author

E-mail: calebepm3@hotmail.com (CPM)

Abstract

Food hoarding is a common behavior used by a variety of animals to cope with periods of low food availability. At the retreating edge of species' distribution, the stressful environment and unfavourable climate conditions may impose severe costs on hoarding behavior. Since relict populations are hotspots for evolution and adaptation, and considering that food hoarding behavior has a strong evolutionary basis, we decided to evaluate the occurrence of behavioral variability in the amount of food cached by the endangered Mount Graham red squirrel (*Tamiasciurus fremonti grahamensis*). We tested the variation in cache size in response to microclimate, soil relief, vegetation, food availability and squirrel sex. The number of pits excavated by squirrels to cache cones was used as a proxy of cache size and was affected by mountain slope aspect and density of trees. More pits were excavated in the northeast facing slopes. The density of trees negatively affects the cache volume on southwest slopes, but not on northeast slopes. The sex of the resident squirrel also affects the number of pits in the squirrel midden, with males excavating 47% more pits than females. Males and females also presented different responses to the mountain slope aspect, with females excavating more pits on northeastern slopes than on southwestern slopes, whereas male cache size did not vary with the slope aspect. Finally, the squirrel's caching behavior did not vary in response to midden microclimate variation, a result with possible implications for the survival of the Mt Graham red squirrels, given the predicted temperature increases in the region due to climate change.

KEYWORDS: behavioral variation, cache size, climate, relict population, *Tamiasciurus fremonti grahamensis*

Introduction

In response to both predictable and unpredictable variation in food availability, several taxa (i.g. carnivores, rodents, corvids and birds of prey) make use of caching behavior to guarantee the food supply during periods of scarcity [1,2]. Caching behavior dampens the variations in food availability, "transferring" part of the resources from the period of abundance to be used during scarcity. Since the behavior is used by an array of different taxonomic groups, considerable variation exists in the type of food stored, amount, duration, substrate in which the food is cached, number and spatial distribution of caches [1,3]. For this reason, model organisms of several different taxa are needed to explore the general patterns and ecological implications of cache behavior.

Squirrels have long served as model organisms in the study of caching behavior in mammals, and have helped us to understand the cognitive mechanisms behind animal decision making, including which criteria are used to decide whether to consume or cache a food item [4] and how far to move a food item before caching it [5]. As result, the observed general pattern is that large food items are preferable options to cache [4] as well as items which are less susceptible to spoilage [3]. More valuable food items are usually dispersed farther from the food source to avoid conspecific pilferage [4], and feeding efficiency is an important factor for rodents, shaping not only their behavior but also morphology and evolution [6,7].

The North American red squirrel (*Tamiasciurus hudsonicus*) uses both larder and scatter-hoarding strategies according to cache site conditions [8]. The cache size is also adjusted to match future energy demand and differs between sexes, due to the differential energetic inventstment in the mating strategies [9]. For red squirrels, cache behavior is important for winter survival, but is also energetically expensive and time consuming [9], exposing the animal to predation risk [10]. The cache size must guarantee winter food

security, but should not be large to the point where food is lost to spoilage [11]. Since the red squirrel cache depends on cold and moist condition for preservation [12], the behavior cost-benefit is also affected by the climate.

For populations at the edges of the species distribution, the harsh climatic conditions, often exacerbated by climate change, can severely disrupt the cache behavior effectiveness by increasing food spoilage [13]. Although disruptions in the cache behavior can be sometimes compensated by behavioral shifts, these behavioral shifts can also drive populations to extinction [13]. Indeed, behavioral shifts in response to changes in environmental factos, both due to natural or anthropogenic causes, can be benefic and allow the populations to persist and thrive in adverse environments, but can also be counteradaptative and create evolutionary traps, which can drive populations to extinction [14,15]. Since relict populations at the edges of the species distribution were gradually exposed to what is now a challenging and changing environment, and were selected from what once was the species core population, they are evolutionary hotspots [16,17], and therefore, excellent models to explore the effect of behavioral shifts on population survival.

This way, the relict population of red squirrels (*Tamiasciurus fremonti grahamensis* J. A. Allen, 1894) in the Mt. Graham, Arizona-U.S.A., presents an extraordinary opportunity to explore the occurrence of behavioral shifts in response to a harsh environment. The Mt. Graham red squirrel is a subspecies in the North American red squirrel complex (*Tamiasciurus hudsonicus, T. fremonti*) [18] isolated from the species core distribution for at least 11 million years [19]. The only population of this subspecies, with around 274 individuals during the study period [20], is severely threatened by the low habitat quality, insect outbreaks, exotic species and human activities [19]. Forest fires are also a threat, with the population being reduced to about

35 individuals in 2017 due to a large forest fire, and recovering to 67 squirrels in 2018 (J Koprowski, personal communication, 2018). In addition to all of these threats, this is the southernmost population of the species and is directly threatened by climate change, as they rely on cold and humid climate for the preservation of cones collected during the fall and consumed during the winter (Finley 1969; Koprowski et al. 2013), and the region temperature is predicted to rise 1.5°C until 2100 [22].

Since behavior is among the most plastic traits of a species and modulates the species interactions with the environment, behavioral shifts are usualy the first adaptative reponses to appear in response to environmental challenges [23]. The main objective of this study was to evaluate the occurrence of behavioral shifts in the caching behavior of the Mount Graham red squirrel (*Tamiasciurus fremonti grahamensis*) in response to microclimate variation. More specifically, we evaluated: 1- If climatic variables have effects on the squirrel's cache size, since climate change can interfere in the cost-benefit of food caching behavior [1] and a fine-tuning of the cache volume reduces energy waste [11]; 2- If relief variables such as altitude, slope declivity and aspect, have effects on the squirrel's cache size, since these relief variables affect the local microclimate [24]; 3- If food abundance has effects on the squirrel's cache size, since a higher food availability reduces the cost to find and transport the cones to the cache, increasing the overall cost-benefit of the cache behavior.

Based on these assumptions, we expect to observe bigger caches in colder areas, where the conditions to cone preservation is better and spoilage is expected to be reduced, increasing the behavior cost-benefit. We also expect to observe bigger caches in areas with colder microclimates, such in higher elevations and in slopes facing the northeast. Finaly, we expect to observe bigger caches in areas with higher cone availability.

Materials and Methods

The study was in the highest elevations of Mount Graham, in the Pinalenõ Mountains, Arizona, USA, between 2770 and 3270 m of altitude, an area with rugged relief, which promotes considerable microclimatic variation in a small geographic area, as part of the Madrean Archipelago. The Madrean Archipelago is a mid-latitude sky island complex, where species of temperate zone are surrounded by a low altitude inhospitable matrix. The montane islands have high biodiversity, each one with its own set of relict populations that have persisted since the end of the last glaciation 10 to 15 k.y.a [21,25]. In fact, with the increase of ~5 ºC in mean annual temperature since the last Glacial maximum, 19 to 23 k.y.a. [26] and the consequent poleward displacement of the distribution of the species, at least 80 populations, including the red squirrels, inhabiting the Madrean Archipelago are the actual edges of the respective species [25]. The study was conducted under permits from the Arizona Game and Fish Department (permit number SP696903), the US Fish and Wildlife Endangered Species (permit number TE041875), and the University of Arizona's Institutional Animal Care and Use Committee (permit number #14-504).

To evaluate the effect of climate on Mt. Graham red squirrel caching behavior, we selected 40 occupied middens distributed along different altitudes, relief, and mountain aspect. Since the subspecies geographic range is extremely restricted, these middens were enough to cover the local habitat variability. We visited each midden 6 times between the fall and winter of 2015 to measure variables of weather, relief, plant structure and estimate the volume of cones stored by the resident squirrels (response variable). During the visits, we also record the sex of the resident squirrels, by visual inspection using binoculars.

To estimate the volume of cones cached by each squirrel, we took advantage of the fact that the animals do not cache the cones loose in the scale pile but deposit them

into holes, here called “pits”, excavated in the scale pile or in the soil (Finley 1969). Thus, we used the number of pits, counted during the cache season, as proxy of the volume of cones cached in each midden. This method follows Gurnell (1984), but in the present study we did not excavate the pits to count the number of cones inside the pits, since it would disturb the midden of these endangered squirrels. The cache size estimates occurred between August 24 and September 12, an interval short enough to avoid any bias caused by the accumulation of cones along the cache season (LM, β = 0.03758, t = 1.726, p-value = 0.096). We also recorded the presence of dead logs near middens, because they are occasionally used to store cones (Finley 1969), but since the presence of logs had no detectable effects on the preliminary results, we excluded the variable from the analysis.

For the weather variables, we use a handheld weather meter (Kestrel 3000) to measure the air temperature (°C) and relative humidity (%), and a digital soil thermometer (HANNA HI45-30) to record soil temperature, the temperature inside of the scale pile, and inside of the pits (average value obtained from 3 randomly selected pits for each midden). Since we could measure only one midden at a time, the dial temperature variation, as well as the variation along different days, could easly mask any difference in middens microclimate. To make the measurements comparable, we used the data recorded by a meteorological station located at the top of the mountain (MGIO-Mt Graham Summit KAZSAFFO4) and by a data-logger (HOBO U23 Pro v2) buried in the ground as a baseline to allow comparison between middens. The weather station was used as baseline for the air temperature and humidity whereas the buried data-logger was used as baseline for the temperatures of soil, pits and scale piles. Since both baselines are fixed in space, by subtracting the baseline values from the values recorded in the middens, taken at the same time, the resultant value is the difference between the midden and the baseline,

which is a value comparable between middens. This way, the temperature and humidity variables are the mean of the values measured and corrected, for each midden, during the 6 visits.

Autumn is a critical period for the storage of cones because it is warmer and drier compared to the microclimate under the snow over winter, thus it is important to keep the cones in cool and humid conditions to avoid spoilage before the snow fall. Since the middens are usually cooler than the soil and air temperature during the fall (mostly due to evaporative heat loss) we calculated an additional variable called "pit cooling effect". This variable was calculated by subtracting the air temperature from the pit temperature, and represents how much cooler is the pit interior compared with the external environment.

Since some species are reported to cache more in areas with longer snow cover periods [2], and considering that memory and learning are important factors that modulate species behavioral [28], we decided to test the effect of snow cover duration on cache size. To do so, we capitalized on the fact that in 2015 all middens were covered by snow on December 12, and so we used the percentage of soil still covered by snow within a 5-m radius of each midden between 10 and 12 of March of 2016 as a proxy for the speed of snowmelt at each midden. We decided to use data from the 2015-2016 winter based on the premise that snow melting speed does not vary randomly along the mountain, but follows a pattern defined by relief and microclimatic factors [29]. Thus, a total of seven microclimatic variables were tested in the present work (Table 1; S2 Dataset).

Table 1. Explanatory variables.

Variables	Type	Unit
Altitude	Relief	Meters

Declivity	Relief	Degrees
Aspect*	Relief	Bearing
Soil temperature*	Microclimate	°C
Air temperature*	Microclimate	°C
Air humidity	Microclimate	°C
Pit temperature	Microclimate	°C
Scale pile temperature	Microclimate	°C
Pit cooling effect	Microclimate	°C
Snow melt speed	Microclimate	Percentage
Forest cover	Forest structure	Percentage
Tree density*	Forest structure	Ind/ha
Live tree density	Forest structure	Ind/ha
Dead tree density	Forest structure	Ind/ha
Live conifer density	Food availability	Ind/ha
Engelmann spruce	Food availability	Ind/ha
Douglas Fir	Food availability	Ind/ha
Ponderosa pine	Food availability	Ind/ha
Mushroom productivity	Food availability	Grams
Resident squirrel sex	Sexual	Binary

List of tested explanatory variables with the respective units. The principal explanatory variables, marked by a *, were selected by PCA analysis and were used to create models for subsequent model selection analysis.

To record the topographic relief variation, we used a GPS unit to record the altitude of each midden, a clinometer to record the declivity and a compass to record the aspect. For vegetation variables, between between 10 and 12 August, we used a densitometer to record the forest cover for each midden and recorded the arboreal community with diamater at breast height (DBH) ≥ 10 cm by using 4 transects of 30x5 meters, starting from the midden in the four cardinal directions. The density of live conifers in the transects was used as a proxy for the availability of cones near each midden. This proxy is based on the premise that the mean cone production of the conifer

species did not vary across the study area, which was tested and confirmed by the Moran's I test for spatial autocorrelation, using data from cone counting for 73 trees of the 3 species that produced cone crops in 2015 (Engelmann spruce: n= 33, p-value= 0.597; Douglas fir: n=35, p-value= 0.59; Ponderosa pine: n= 5, p-value= 0.269), nor was correlated with mountain aspect, which was tested and confirmed by linear models (Engelmann spruce: β = 0.694, t = 1.109, p = 0.276; Douglas fir: β = 0.001, t = 0.389, p = 0.7; Ponderosa pine: β = -0.008, t = -0.695, p = 0.537). From the tree transect data, we also calculated the trees density within the midden vicinity, density of live trees, density of dead trees, density of Englemann spruce, Douglas fir and Ponderosa pine, which are the main tree species that produced cones.

Between 3 and 7 August, we also estimate the productivity of mushrooms, an alternative food resource used and cached by squirrels [19,30], and their availability may interfere with the behavior of the animals. Mushroom productivity, recorded in grams, was estimated through 4 transects of 10x1 m, starting from the midden in the four cardinal directions. All mushrooms of all species known to be consumed by the squirrels [30,31] were collected and weighed.

To analyze the data, we verified the normality of the variables, using the Shapiro-Wilk test, and normalized the variables when needed. The response variable, number of pits, was log transformed to fulfill normality requirements, and the slope aspect, a circular variable, was tested for uniformity using a Kuiper uniformity test [32]. To allow the slope aspect variable to be analyzed together with the other linear variables, we linearized the variable by dividing it along the northeast-southwest axis (bearings 45° and 225°, where north is 0°\360°), and transfering the records in the northwest semicircle to the southwest semicircle. This way, the resultant semicircle can be treated as a linear variable, ranging from bearing 45° to 225°, where 45° represents a place where the soil slope is facing the

northeast, whereas 225° represents a place where the soil slope is facing towards southwest. We decided to use a northeast-southwest axis due visual and preliminary inspections of the raw data, which pointed to maximal variation along the northeast-southwest axis.

Due to the large number of explanatory variables, to avoid type I statistical errors, we divided the variables ~~in~~ biotic and abiotic and used Principal Component Analysis (PCA) to identify and delete the variables with high similarity within each group [33]. In this process, we used the PCA results for each group, selecting the more dissimilar variables based on the dissimilarity values within the first two dimensions, testing whether the variables were correlated with other similar variables, and then, deleting the correlated variable with smaller dissimilarity values. We performed a PCA again with the remaining biotic and abiotic variables, repeating the process to keep only the most dissimilar explanatory variables without correlations. After reducing the number of explanatory variables, we used a multiple competing hypothesis approach [34] to evaluate the effect of these remaining variables on the number of pits excavated by the squirrels.

For the competing hypothesis approach, we created a set of Generalized Linear Models (GLM), in which each model uses a different combination of one or two explanatory variables to explain the response variable (number of pits). For the model with two explanatory variables, we tested for both summatory and interaction effects between the explanatory variables, however, since interaction models performed poorly when compared to summation models, they were removed of the analisys. We did not test models with more than two variables due to the modest sample size of the dataset. A null model was created by using aleatory generated numbers as an explanatory variable, and the Akaike Information Criterion corrected ~~to~~ small sample size was used to compare the models [34]. For each model, we calculated the wAICc, a parameter of the relative

likelihood, and the ΔAICc, a parameter of relative difference between models. Models with ΔAICc < 2 were considered equally plausible. To reinforce the analysis, we calculated frequency πi, which is a bootstrap method to calculate the frequency in which a model *i* is selected as the best model in the set in 10000 random resamples of the dataset [34].

Finally, due to differences in reproductive strategy between males and females, the peak of reproductive energy expendure occurs in early spring for males and late spring for females [9]. Considering that in early spring, the males rely on cached food to maintain energy demanding reproductive activities, we tested the effect of sex of the resident squirrel on the cache size using Student's t-test and linear model regression. All statistics were performed using the R software [35] software, using the packages "circular" [36], "bbmle" [37], "FactoMineR" [38], "akima" [39], "plotrix" [40] and "ape" [41].

Results

From the 40 selected middens, 12 were excluded due to migration/death of the resident squirrel during the study. From the 28 remaining, 15 were occupied by males, 11 by females and 2 animals of unknown sex. The 28 remaining middens were evenly distributed in all aspects of the mountain slopes (Kuiper's Test of Uniformity, $p > 0.15$).

The PCA analysis reduced the 19 initial explanatory variables (excluding the resident squirrel sex, which is a binary variable) to only 4 principal explanatory variables, which are: air temperature, soil temperature, aspect and the density of trees in the midden vicinity (S1 Fig). The other 3 vegetation variables, 2 relief variables, 5 climate variables and 5 food availability variables were removed from the analysis due to their correlation with at least one of the four more dissimilar variables. Based on the four selected explanatory variables, eleven models were created, including the null model, and

compared using the Akaike Information Criterion method (Table 2). The model with highest explanatory power included the summed effects of relief aspect and density of trees as predictor variables (Fig 1), with the middens located in the northeastern slopes containing more pits than the middens in the southwestern slopes. At the same time, in the southeastern slopes, the middens surrounded by more trees contained fewer pits than the middens with less trees in its vicinity. This negative correlation between density of trees and number of pits was not observed in the northeast-facing slopes.

Table 2. Variables affecting red squirrel cache size.

Models	ΔAICc	Df	wAICc	πi
GLM10: NP ~ Aspect + Density of Trees	0.0	4	0.7161	0.599
GLM3: NP ~ Aspect	3.4	3	0.1337	0.061
GLM8: NP ~ Aspect+ Air Temperature	4.2	4	0.0865	0.220
GLM6: NP ~ Aspect+ Soil Temperature	5.3	4	0.0518	0.035
GLM5: NP ~ Soil Temperature + Air Temperature	10.3	4	0.0041	0.058
GLM4: NP ~ Density of Trees	11.5	3	0.0022	0.001
GLM9: NP ~ Air Temperature + Density of Trees	11.6	4	0.0021	0.017
GLM7: NP ~ Soil Temperature + Density of Trees	12.4	4	0.0015	0.003
GLM1: NP ~ Soil Temperature	12.9	3	0.0011	0.001
GLM2: NP ~ Air Temperature	14.5	3	<0.001	0.000
GLM0: NP ~ Null (aleatory generated values)	15.1	3	<0.001	0.000

Results of the model selection for Number of Pits (NP) excavated by Mt. Graham red squirrels. We fitted generalized linear models and calculated the corrected Akaike relative difference (ΔAICc), relative likelihood (wAICc) and a bootstrap model selection frequency (πi) to all models. Models with ΔAICc $\leq$ 2 were considered as equally plausible. The πi parameter was calculated based on 10.000 permutations.

Fig 1. Cache size in response to site aspect and tree density. Predicted number of pits excavated by Mt. Graham red squirrels in response to the site aspect (linearized in the x-axis), and density of trees (y-axis), based on the best fitted model. The black points represent the sampled middens.

The best model explained 44% of the observed variation in midden number of pits and received a wAICc = 0.7162, a frequency πi = 0.59, by far the most plausible model

(Table 2). Aspect was clearly the most important variable, since it was also present in the four models with higher explanatory power, while the tree density variable was, by itself, a weak variable and obtained only the sixth position in the rank of better models.

Due to its categorical nature, the resident squirrel sex was not included in the PCA and competing hypothesis analysis, instead, we divided the middens according to the sex of the resident squirrel and compared the two groups with student t-tests. We found differences in the number of pits excavated by males and females (t = 2.59, df = 23.78, p = 0.016), with males digging 47% more pits than females (Fig 2). We found marginal differences in the aspect of middens of males and females (t = -0.105, df = 20.196, p-value = 0.054), with more male squirrels being found on slopes with a northeastern aspect and more females in the southwestern facing areas (Fig 3). We found no evidence of an aspect effect on the cache behavior of males (β = -0.0006, t = -0.603, p = 0.556), whereas for females, the effect of the aspect was strong (β = -0.359, t = -7.877, p << 0.01), with females on northeastern slopes digging more pits than the females on the southwest facing areas (Fig 4).

Fig 2. Cache size of males and females. Differences in the number of pits excavated by male and female red squirrels on Mt. Graham, Arizona, USA.

Fig 3. Cache size along the mountain aspect. Circular distribution of the sampled middens along the mountain aspect. The radial axis represents the number of pits excavated by each resident squirrel. The distance between points in the picture does not represent geographical distances at the study site. The sex of the resident squirrels is indicated by blue points for males, red triangles for females and black diamonds for squirrels with unknown sex.

Fig 4. Differences in the response of males and females to site aspect. Regression of the number of pits excavated by males (blue points) and females (red triangles) in response of the aspect (linearized in the x-axis). For the description of the linearization process of the aspect variable, see the methods section.

Discussion

We found evidence to support our hypothesis about the existence of behavioral variation in the cache behavior of Mount Graham red squirrels, so that the number of pits excavated by these rodents changes according to the site aspect, density of trees and the sex of the resident squirrel. The data did not support the existence of temperature related behavioral variation, as squirrels displayed no response to any of the tested temperature variables. We found support for the existence of behavioral shifts in response to topographic relief, with middens in the northeastern slopes of the mountain containing more pits than in the southwestern slopes. This response was driven by females that strongly responded to aspect, whereas males did not. We found no support for behavioral shifts in response to food availability.

The absence of a response in the squirrel's cache behavior to altitude, with the variable even excluded during the PCA, is not surprising considering that altitude per se (i.e. vertical distance between a point and sea level) did not have causative effects on biological systems. Instead, altitude must be considered as a strong proxy of other environmental variables, such as barometric pressure, temperature and humidity, which in turn have effects on biological systems [24]. Considering that temperature and humidity were also poor predictors of squirrel cache behavior, it is not a surprise that altitude was excluded by the PCA.

The absence of a response evidenced in the squirrel's cache behavior to variation in the temperature along the mountain is surprising given how important temperature is to cone preservation [12,42] and thus to squirrel survival. However, several factors could interfere and/or preclude the emergence and maintenance of such behavioral responses. First, mean air temperature varied only 4.7 °C between middens, it is possible that squirrels simply lack the capability to detect such small variation and thus, do not respond

to these small temperature variations, independent of its importance to cone preservation. Even if squirrels are not capable of perceiving this variation in mean temperature, increases of just a degree or two are enough to impact cone preservation (J Koprowski, unpublished data), and a minimum increase of 1.5 °C is expected due to climate change before 2100 [22].

Another possible explanation is that the squirrels did not respond to the mean temperature, but to the occurrence and duration of extreme temperature events, such as observed in ~~*Perisoreus canadensis*~~ [13]. For food preservation, one or few events of extreme temperatures may cause cache spoilage through frost thaw or, in the case of red squirrels, by cone dehydration [12,13]. However, extreme temperature events were not detectable by the current experiment design, and therefore, a more specific experiment would be required to clarify this issue. This is a question with high conservation value, since it relates to whether the Mt. Graham red squirrels have behavioral ways to deal with the climate change predicted to the region [22].

The present study observed the effect of the site aspect, density of trees and sex of the resident squirrel on cache size, however, it was not possible to completely isolate and precisely estimate the effect of each one of these three variables on the animals' cache behavior. The isolation and evaluation of these three effects was hindered by the unexpected unequal distribution of males and females in the montain slopes, along the Notheast-Southwest axis. The loss of 30% of the middens due to squirrel mortality/migration during the sampling period further reduced the sample size and therefore limiting the statistical options available for data corrections. To precisely estimate the effect of aspect, density of trees and squirrel sex on cache size, a study with standardized sample size for males and females along the mountain aspect is

recommended, using an initial sample size big enough to compensate the low annual survival rate of Mt Graham red squirrels [20].

Althouth the exact mechanisms underlying the observed results are still not clear, there are some possible explanations that fit the observed data. A possible mechanism driving the effect of both aspect and density of trees on cache size is the incidence of solar radiation. In the northern-hemisphere, the southern mountain slopes receive sunlight at a more perpendicular angle than the northern slopes, and the forest canopy blocks more solar radiation from reaching the ground when the light came from a less perpendicular angle, such on the northern slopes [43]. Together, aspect and density of trees cause the northern slopes to be more shaded than southern slopes. This way, solar radiation might explain why cache size varies along the northeast-southwestern axis of the aspect, as observed for female squirrels, but its consequences on the squirrel cache are not clear.

Another possible mechanism underlying the observed differences in responses of males and females to tree density is related to the individual's perception of predation risk. Differences in boldness, vigilance and risk perception between sexes are a relatively common phenonema [44], and vigilance activities do interfere with foraging efficiency [45]. Since avian predation is a significant cause of mortality in the Mt. Graham squirrel population [10,20] and females have a higher annual survival rate than males [20], it is possible that tree density has different effects on risk perception for males and females, affecting their respective cache efficiency, effort and therefore cache size.

Our results document the existence of cache related behavioral variation in response to the topographic relief, density of trees and individual sex, in an endangered red squirrel subspecies. Although the mechanism by which these variables affect squirrel cache behavior are still not clear, it may be unrelated to site temperature, as would be usually expected based on the literature. Since temperature is important to cone

preservation [12], and the Mt. Graham red squirrels did not appear to behaviorally respond to temperature, the future increase in temperature may turn the ~~Pinalenõ~~ Mountains into unsuitable forest for these red squirrels. In the face of already unavoidable climate change, sky island complexes, such as the Madrean Archipelago [25,46], can be used as natural laboratories, providing us the opportunity to better understand how species characteristics, including behavior, affects its persistence capability. This may also allow us to develop and improve our conservation methods to protect populations on the edge of their distributions and on the edge of extinction.

Acknowledgements

We would like to thank to the Conservation Research Laboratory of the University of Arizona, Arizona Agriculture Experiment Station and the USDA Forest Service for logistic and financial support, to the Coordenação de Aperfeiçoamento de Pessoal de Nível Superior (CAPES) and Universidade Estadual Paulista "Júlio de Mesquita Filho" (UNESP-RC) for providing a one-year studentship at the study site, and to the Conselho Nacional de Desenvolvimento Científico e Tecnológico (CNPq) for a studentship in Brazil.

References

1. Sutton AO, Strickland D, Norris DR. Food storage in a changing world: implications of climate change for food-caching species. Clim Chang Responses. 2016;3: 12. doi:10.1186/s40665-016-0025-0
2. Wall SB Vander. Food hoarding in animals. University of Chicago Press; 1990.
3. Hadj-Chikh LZ, Steele MA, Smallwood PD. Caching decisions by grey squirrels:

a test of the handling time and perishability hypotheses. Anim Behav. 1996;52: 941–948. Available: http://psycnet.apa.org/psycinfo/1996-06811-009

4. Preston SD, Jacobs LF. Mechanisms of cache decision making in fox squirrels (*Sciurus niger*). J Mammal. 2009;90: 787–795. doi:10.1644/08-MAMM-A-254.1
5. Tamura N, Hashimoto Y, Hayashi F. Optimal distances for squirrels to transport and hoard walnuts. Anim Behav. 1999;58: 635–642.
6. Smith CC. The coevolution of pine squirrels (*Tamiasciurus*) and conifers. Ecol Monogr. 1970;40: 349–371. doi:10.2307/1942287
7. Cox PG, Rayfield EJ, Fagan MJ, Herrel A, Pataky TC, Jeffery N. Functional evolution of the feeding system in rodents. PLoS One. 2012;7: e36299. doi:10.1371/journal.pone.0036299
8. Hurley T a, Robertson RJ. Variation in the food hoarding behaviour of red squirrels. Behav Ecol Sociobiol. 1990;26(2): 91–97.
9. Archibald DW, Fletcher QE, Boutin S, McAdam AG, Speakman JR, Humphries MM. Sex-specific hoarding behavior in North American red squirrels (*Tamiasciurus hudsonicus*). J Mammal. 2013;94: 761–770. doi:10.1644/12-MAMM-A-213.1
10. Schauffert CA, Koprowski JL, Greer VL, Alanen MI, Hutton KA, Young PJ. Interactions between predators and Mt. Graham red squirrels (*Tamiasciurus hudsonicus grahamensis*). Southwest Nat. 2002;47: 498. doi:10.2307/3672515
11. Gendron RP, Reichman OJ. Food perishability and inventory management: a comparison of three caching strategies. Am Nat. 1995;145: 948–968. Available: http://www.jstor.org/stable/2463174
12. Finley Jr RB. Cone caches and middens of *Tamiasciurus* in the Rocky Mountain region. Misc Publ Museum Nat Hist Univ Kansas. 1969;51: 233–273. Available:

http://scholar.google.com/scholar?hl=en&btnG=Search&q=intitle:Cone+caches+and+middens+of+Tamiasciurus+in+the+rocky+mountain+region#0

13. Waite TA, Strickland D. Climate change and the demographic demise of a hoarding bird living on the edge. Proc R Soc B Biol Sci. 2006;273: 2809–2813. doi:10.1098/rspb.2006.3667

14. Robertson BA, Rehage JS, Sih A. Ecological novelty and the emergence of evolutionary traps. Trends Ecol Evol. 2013;28: 552–560. doi:10.1016/j.tree.2013.04.004

15. Gross K, Pasinelli G, Kunc HP. Behavioral plasticity allows short-term adjustment to a novel environment. Am Nat. 2010;176: 456–464.

16. Hampe A, Jump AS. Climate relicts: past, present, future. Annu Rev Ecol Evol Syst. 2011;42: 313–333. doi:10.1146/annurev-ecolsys-102710-145015

17. Lesica P, Allendorf FW. When are peripheral populations valuable for conservation? Conserv Biol. 2015;9: 753–760.

18. Hope AG, Malaney JL, Bell KC, Salazar-Miralles F, Chavez AS, Barber BR, et al. Revision of widespread red squirrels (genus: *Tamiasciurus*) highlights the complexity of speciation within North American forests. Mol Phylogenet Evol. 2016;100: 170–182. doi:10.1016/j.ympev.2016.04.014

19. Granillo KA, Froehlich GF. Mt. Graham red squirrel natural history and pre-observatory construction conservation efforts. In: Sanderson HR, Koprowski JL, editors. The last refuge of the Mt Graham red squirrel. The University of Arizona Press; 2009.

20. Goldstein EA, Merrick MJ, Koprowski JL. Functional semelparity drives population dynamics and endangers a peripheral population. Biol Conserv. 2017;205: 52–59. doi:10.1016/j.biocon.2016.11.017

21. Koprowski JL, Doumas SL, Merrick MJ, Oleson B, Posthumus EE, Jessen TG, et al. It's lonely at the top: biodiversity at risk to loss from climate change. Merging science and management in a rapidly changing world: Biodiversity and management of the Madrean Archipelago III and 7th Conference on Research and Resource Management in the Southwestern Deserts. Tucson, AZ, USA; 2013. pp. 53–59.

22. IPCC. Climate change 2014: Synthesis report. Contribution of working groups I, II and III to the Fifth assessment report of the Intergovernmental Panel on Climate Change. Geneva, Switzerland; 2014. doi:10.1017/CBO9781107415324

23. Wong BBM, Candolin U. Behavioral responses to changing environments. Behav Ecol. 2015;26: 665–673. doi:10.1093/beheco/aru183

24. Körner C. The use of "altitude" in ecological research. Trends Ecol Evol. 2007;22: 569–574. doi:10.1016/j.tree.2007.09.006

25. Warshall P. The Madrean Sky Island Archipelago: a planetary overview. In: DeBano LH, Ffolliott PH, Ortega-Rubio A, Edminster GJG, Hamre RH, B. C, editors. Biodiversity and management of the Madrean Archipelago: The sky islands of Southwestern United States and Northwestern Mexico. Tucson, AZ, USA: Fort Collins, CO: U.S. Department of Agriculture, Forest Service, Rocky Mountain Forest and Range Experiment Station; 1995. pp. 6–18.

26. Correa-Metrio A, Bush M, Lozano-García S, Sosa-Nájera S. Millennial-scale temperature change velocity in the continental northern neotropics. PLoS One. 2013;8: 1–11. doi:10.1371/journal.pone.0081958

27. Gurnell J. Home range, territoriality, caching behaviour and food supply of the red squirrel (*Tamiasciurus hudsonicus fremonti*) in a subalpine lodgepole pine forest. Anim Behav. 1984;32: 1119–1131. doi:10.1016/S0003-3472(84)80228-6

28. Mery F, Burns JG. Behavioural plasticity: an interaction between evolution and experience. Evol Ecol. 2010;24: 571–583. doi:10.1007/s10682-009-9336-y

29. Abudu S, Sheng Z ping, Cui C liang, Saydi M, Sabzi HZ, King JP. Integration of aspect and slope in snowmelt runoff modeling in a mountain watershed. Water Sci Eng. 2016;9: 265–273. doi:10.1016/j.wse.2016.07.002

30. Hendricks P, Hendricks LM. Use of conifers by red squirrels (*Tamiasciurus hudsonicus*) in Montana for drying and caching mushrooms. Northwest Nat. 2015;96: 240–242. doi:https://doi.org/10.1898/1051-1733-96.3.240

31. Koprowski JL, Greer VL, Merrick MJ. Mt. Graham red squirrel monitoring program - 2015 Annual report. Tucson, AZ, USA; 2015.

32. Pewsey A, Neuhäuser M, Ruxton GD. Circular statistics in R. Oxford University Press; 2013.

33. Jolliffe IT. Principal Component Analysis [Internet]. 2nd ed. New York: Springer-Verlag; 2002. doi:10.1007/b98835

34. Burnham KP, Anderson DR. Model selection and multi-model inference: a practical information-theoretic approach. 2nd ed. Springer; 2010.

35. R Development Core Team. R: A language and environment for statiscal computing [Internet]. Vienna, Austria: R Foundation for Statistical Computing; 2016. Available: http://www.r-project.org

36. Agostinelli C, Lund U. R package "circular": circular statistics [Internet]. 2011. Available: https://r-forge.r-project.org/projects/circular/

37. Bolker B, R Development Core Team. bbmle: tools for general maximum likelihood estimation. R package version 1.0.20. 2017.

38. Le S, Josse J, Husson F. FactoMineR: an R package for multivariate analysis. J Stat Softw. 2008;25: 1–18. doi:10.18637/jss.v025.i01

39. Akima H, Gebhardt A. akima: interpolation of irregularly and regularly spaced data. R package version 0.6-2. 2016.

40. Lemon J. Plotrix: a package in the red light district of R. R-News. 2016. p. R-News, 6(4): 8-12.

41. Paradis E, Schliep K. ape 5.0: an environment for modern phylogenetics and evolutionary analyses in R. Bioinformatics; 2018.

42. Smith AA, Mannan RW. Distinguishing characteristics of Mount Graham red squirrel midden sites. J Wildl Manage. 1994;58: 437–445. doi:http://dx.doi.org/10.2307/3809314

43. Courbaud B, De Coligny F, Cordonnier T. Simulating radiation distribution in a heterogeneous Norway spruce forest on a slope. Agric For Meteorol. 2003;116: 1–18. doi:10.1016/S0168-1923(02)00254-X

44. Elgar M. Predator vigilance and group size in mammals and birds: a critical review of the empirical evidence. Biol Rev. 1989;64: 13–33. doi:10.1111/j.1469-185X.1989.tb00636.x

45. Verdolin JL. Meta-analysis of foraging and predation risk trade-offs in terrestrial systems. Behav Ecol Sociobiol. 2006;60: 457–464. doi:10.1007/s00265-006-0172-6

46. Parmesan C. Ecological and evolutionary responses to recent climate change. Annu Rev Ecol Evol Syst. 2006;37: 637–669. doi:10.1146/annurev.ecolsys.37.091305.110100

Supporting Information Captions

S1 Figure: Principal Component Analysis. PCA, showing the multivariate variation of the biotic (a) and abiotic (b) explanatory variables. Variables with similar variation were tested for correlation by the Pearson's correlation coefficient, when correlations were found, the most dissimilar variable was kept while the other was discarded. The resulting biotic and abiotic variables were put all together and the process was repeated (c). In the end pro the process, only the four variables (tree density, soil temperature, air temperature and aspect in a Northeast-southwestern axis) remained and therefore were used in the model selection.

S2 Dataset: Complete dataset. .CSV file containing the data used in the present study.

Figure 1

Click here to access/download;Figure;Figure1_hd.tif

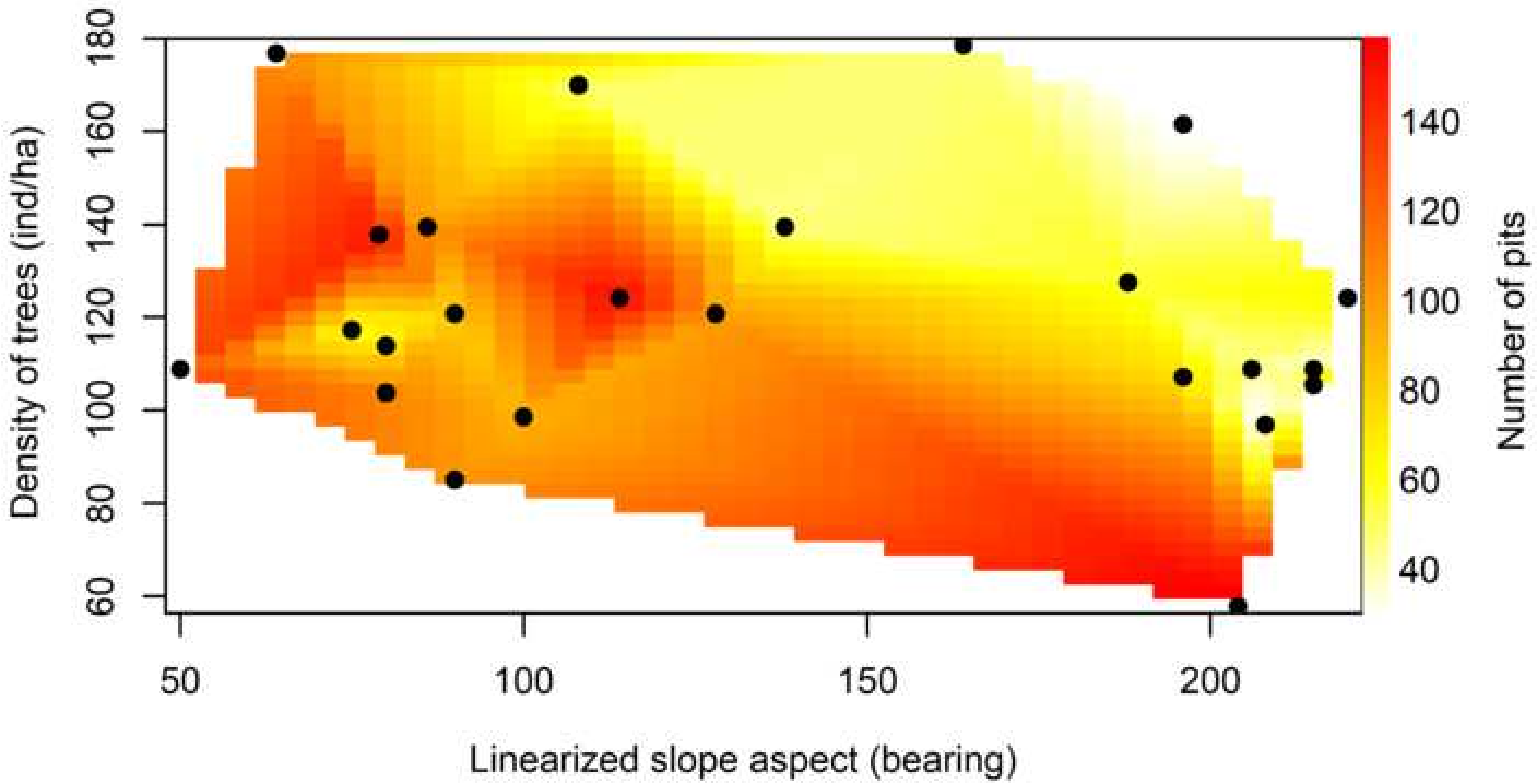

Figure 2

Click here to access/download;Figure;Figure2_hd.tif

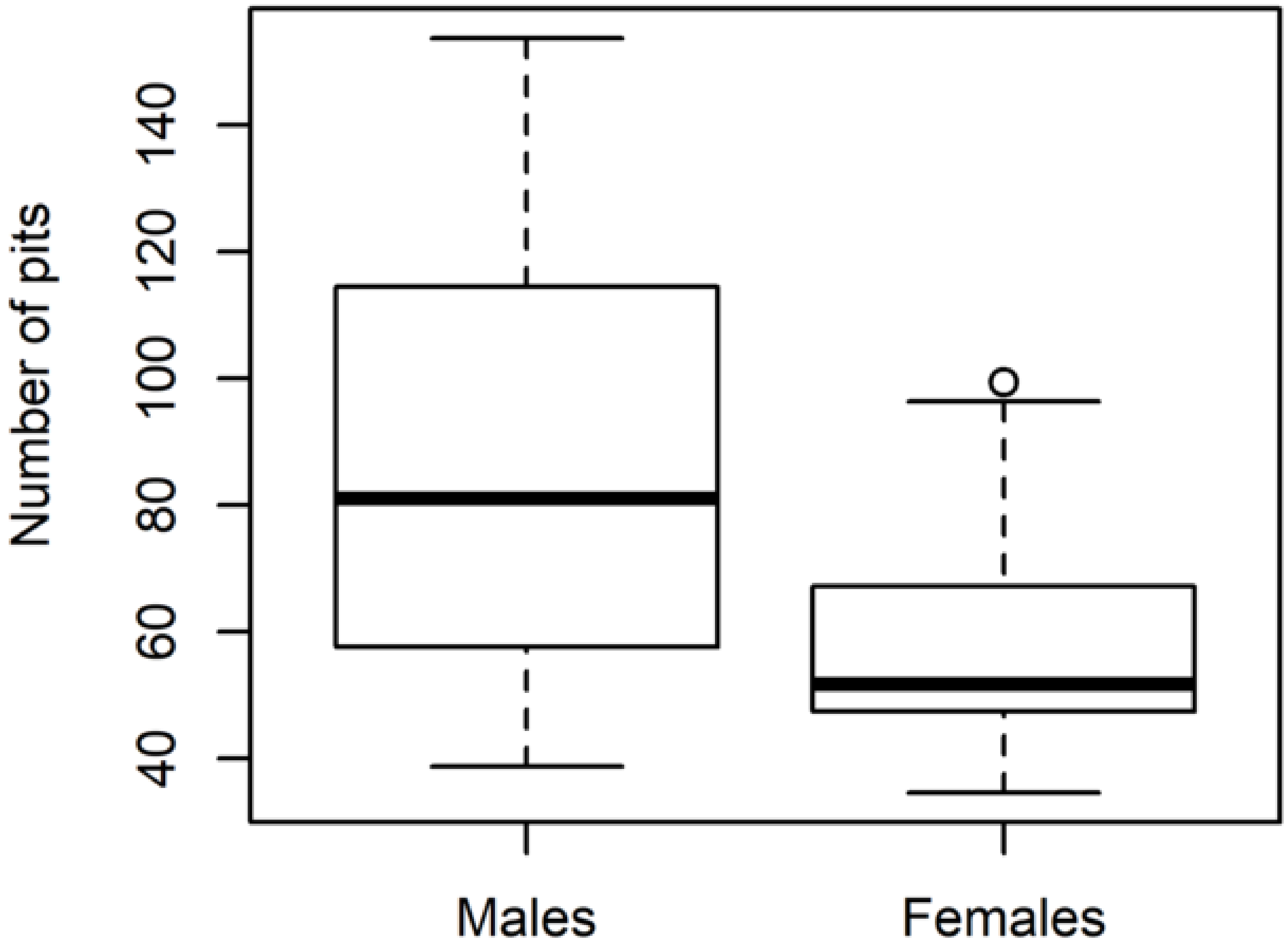

Figure 3

Click here to access/download;Figure;Figure3_hd.tif

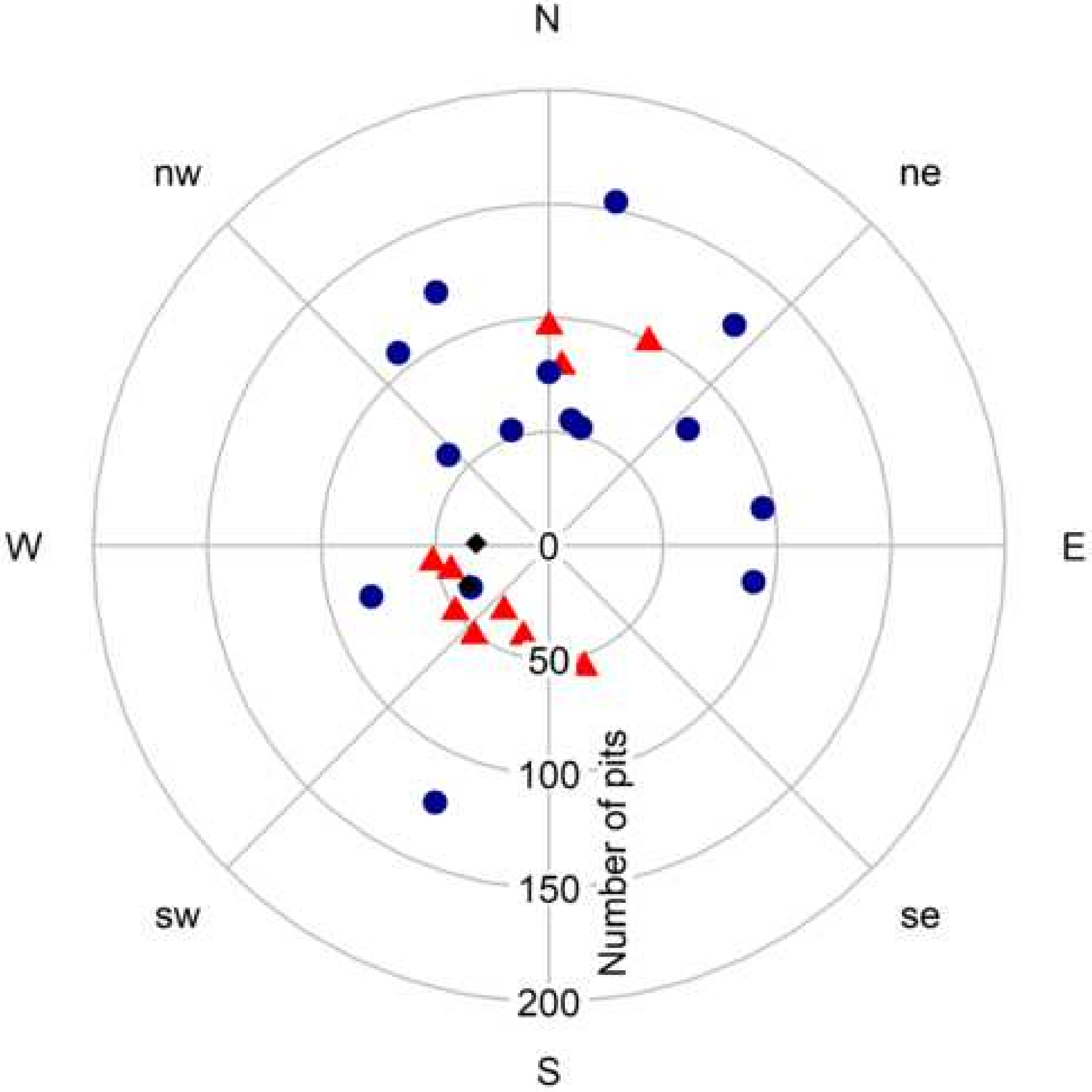

Figure 4

Click here to access/download;Figure;Figure4_hd.tif

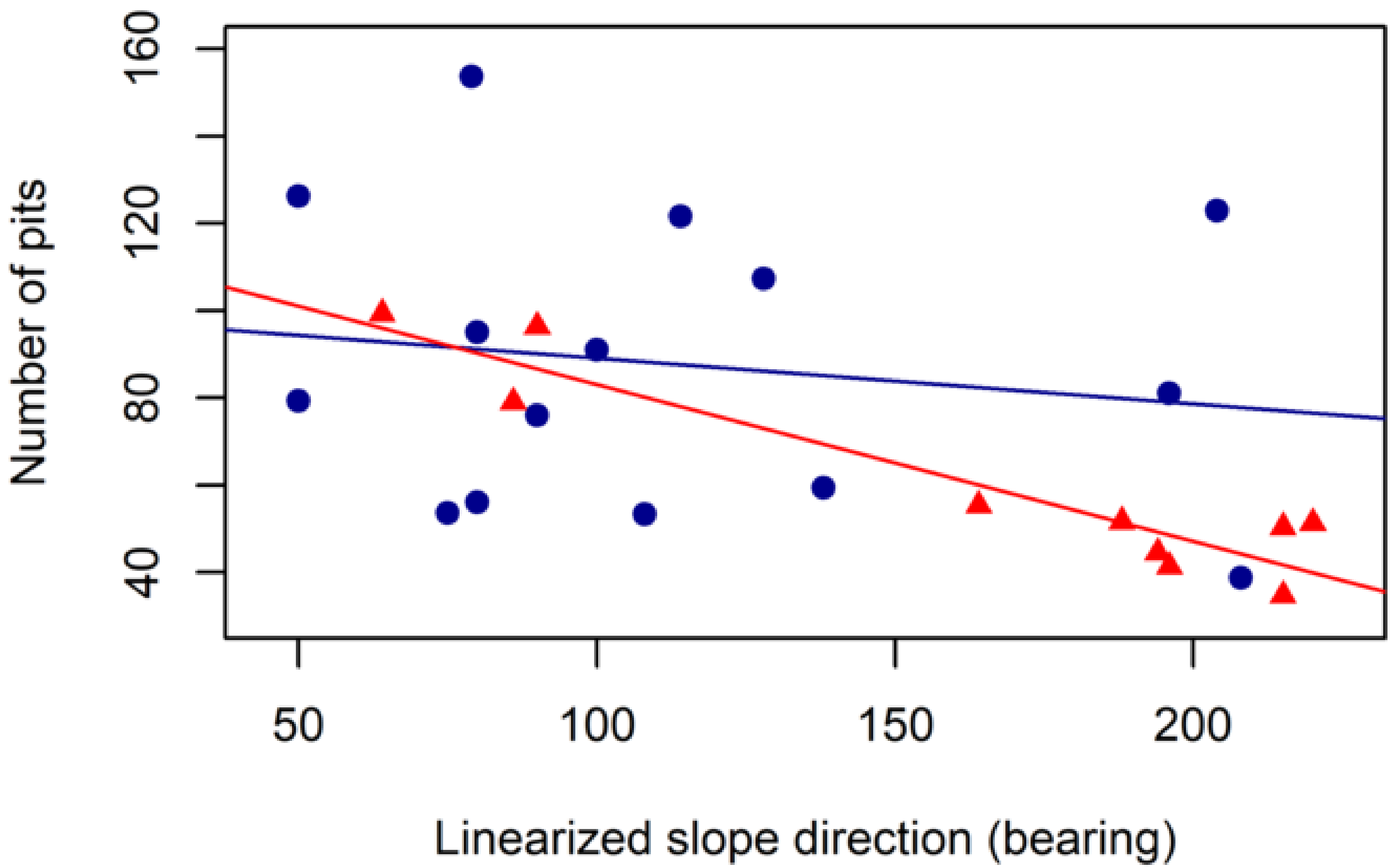

Click here to access/download
Supporting Information
Supporting information.docx

Click here to access/download
Supporting Information
Dataset.csv

Does caching strategy vary with microclimate in endangered Mt. Graham red squirrels?

Calebe Pereira Mendes[1]*, and John Koprowski[2]

[1] *Laboratório de Biologia da Conservação, Instituto de Biociências, Universidade Estadual Paulista "Júlio Mesquita Filho" (UNESP), Rio Claro, São Paulo*, Brazil.

[2] *School of Natural Resources and the Environment, University of Arizona, Tucson, Arizona, United States of America.*

* Corresponding author

E-mail: calebepm3@hotmail.com (CPM)

Abstract

Food hoarding is a common behavior used by a variety of animals to cope with periods of low food availability. At the retreating edge of species' distribution, the stressful environment and unfavourable climate conditions may impose severe costs on hoarding behavior. Since relict populations are hotspots for evolution and adaptation, and considering that food hoarding behavior has a strong evolutionary basis, we decided to evaluate the occurrence of behavioral variability in the amount of food cached by the endangered Mount Graham red squirrel (*Tamiasciurus fremonti grahamensis*). We tested the variation in cache size in response to microclimate, soil relief, vegetation, food availability and squirrel sex. The number of pits excavated by squirrels to cache cones was used as a proxy of cache size and was affected by mountain slope aspect and density of trees. More pits were excavated in the northeast facing slopes. The density of trees negatively affects the cache volume on southwest slopes, but not on northeast slopes. The sex of the resident squirrel also affects the number of pits in the squirrel midden, with males excavating 47% more pits than females. Males and females also presented different responses to the mountain slope aspect, with females excavating more pits on northeastern slopes than on southwestern slopes, whereas male cache size did not vary with the slope aspect. Finally, the squirrel's caching behavior did not vary in response to midden microclimate variation, a result with possible implications for the survival of the Mt Graham red squirrels, given the predicted temperature increases in the region due to climate change.

KEYWORDS: behavioral variation, cache size, climate, relict population, *Tamiasciurus fremonti grahamensis*

Introduction

In response to both predictable and unpredictable variation in food availability, several taxa (i.g. carnivores, rodents, corvids and birds of prey) make use of caching behavior to guarantee the food supply during periods of scarcity [1,2]. Caching behavior dampens the variations in food availability, “transferring” part of the resources from the period of abundance to be used during scarcity. Since the behavior is used by an array of different taxonomic groups, considerable variation exists in the type of food stored, amount, duration, substrate in which the food is cached, number and spatial distribution of caches [1,3]. For this reason, model organisms of several different taxa are needed to explore the general patterns and ecological implications of cache behavior.

Squirrels have long served as model organisms in the study of caching behavior in mammals, and have helped us to understand the cognitive mechanisms behind animal decision making, including which criteria are used to decide whether to consume or cache a food item [4] and how far to move a food item before caching it [5]. As result, the observed general pattern is that large food items are preferable options to cache [4] as well as items which are less susceptible to spoilage [3]. More valuable food items are usually dispersed farther from the food source to avoid conspecific pilferage [4], and feeding efficiency is an important factor for rodents, shaping not only their behavior but also morphology and evolution [6,7].

The North American red squirrel (*Tamiasciurus hudsonicus*) uses both larder and scatter-hoarding strategies according to cache site conditions [8]. The cache size is also adjusted to match future energy demand and differs between sexes, due to the differential energetic invenstment in the mating strategies [9]. For red squirrels, cache behavior is important for winter survival, but is also energetically expensive and time consuming [9], exposing the animal to predation risk [10]. The cache size must guarantee winter food

security, but should not be large to the point where food is lost to spoilage [11]. Since the red squirrel cache depends on cold and moist condition for preservation [12], the behavior cost-benefit is also affected by the climate.

For populations at the edges of the species distribution, the harsh climatic conditions, often exacerbated by climate change, can severely disrupt the cache behavior effectiveness by increasing food spoilage [13]. Although disruptions in the cache behavior can be sometimes compensated by behavioral shifts, these behavioral shifts can also drive populations to extinction [13]. Indeed, behavioral shifts in response to changes in environmental factos, both due to natural or anthropogenic causes, can be benefic and allow the populations to persist and thrive in adverse environments, but can also be counteradaptative and create evolutionary traps, which can drive populations to extinction [14,15]. Since relict populations at the edges of the species distribution were gradually exposed to what is now a challenging and changing environment, and were selected from what once was the species core population, they are evolutionary hotspots [16,17], and therefore, excellent models to explore the effect of behavioral shifts on population survival.

This way, the relict population of red squirrels (*Tamiasciurus fremonti grahamensis* J. A. Allen, 1894) in the Mt. Graham, Arizona-U.S.A., presents an extraordinary opportunity to explore the occurrence of behavioral shifts in response to a harsh environment. The Mt. Graham red squirrel is a subspecies in the North American red squirrel complex (*Tamiasciurus hudsonicus, T. fremonti*) [18] isolated from the species core distribution for at least 11 million years [19]. The only population of this subspecies, with around 274 individuals during the study period [20], is severely threatened by the low habitat quality, insect outbreaks, exotic species and human activities [19]. Forest fires are also a threat, with the population being reduced to about

35 individuals in 2017 due to a large forest fire, and recovering to 67 squirrels in 2018 (J Koprowski, personal communication, 2018). In addition to all of these threats, this is the southernmost population of the species and is directly threatened by climate change, as they rely on cold and humid climate for the preservation of cones collected during the fall and consumed during the winter (Finley 1969; Koprowski et al. 2013), and the region temperature is predicted to rise 1.5°C until 2100 [22].

Since behavior is among the most plastic traits of a species and modulates the species interactions with the environment, behavioral shifts are usualy the first adaptative reponses to appear in response to environmental challenges [23]. The main objective of this study was to evaluate the occurrence of behavioral shifts in the caching behavior of the Mount Graham red squirrel (*Tamiasciurus fremonti grahamensis*) in response to microclimate variation. More specifically, we evaluated: 1- If climatic variables have effects on the squirrel's cache size, since climate change can interfere in the cost-benefit of food caching behavior [1] and a fine-tuning of the cache volume reduces energy waste [11]; 2- If relief variables such as altitude, slope declivity and aspect, have effects on the squirrel's cache size, since these relief variables affect the local microclimate [24]; 3- If food abundance has effects on the squirrel's cache size, since a higher food availability reduces the cost to find and transport the cones to the cache, increasing the overall cost-benefit of the cache behavior.

Based on these assumptions, we expect to observe bigger caches in colder areas, where the conditions to cone preservation is better and spoilage is expected to be reduced, increasing the behavior cost-benefit. We also expect to observe bigger caches in areas with colder microclimates, such in higher elevations and in slopes facing the northeast. Finaly, we expect to observe bigger caches in areas with higher cone availability.

Materials and Methods

The study was in the highest elevations of Mount Graham, in the Pinalenõ Mountains, Arizona, USA, between 2770 and 3270 m of altitude, an area with rugged relief, which promotes considerable microclimatic variation in a small geographic area, as part of the Madrean Archipelago. The Madrean Archipelago is a mid-latitude sky island complex, where species of temperate zone are surrounded by a low altitude inhospitable matrix. The montane islands have high biodiversity, each one with its own set of relict populations that have persisted since the end of the last glaciation 10 to 15 k.y.a [21,25]. In fact, with the increase of ~5 ^{o}C in mean annual temperature since the last Glacial maximum, 19 to 23 k.y.a. [26] and the consequent poleward displacement of the distribution of the species, at least 80 populations, including the red squirrels, inhabiting the Madrean Archipelago are the actual edges of the respective species [25]. The study was conducted under permits from the Arizona Game and Fish Department (permit number SP696903), the US Fish and Wildlife Endangered Species (permit number TE041875), and the University of Arizona's Institutional Animal Care and Use Committee (permit number #14-504).

To evaluate the effect of climate on Mt. Graham red squirrel caching behavior, we selected 40 occupied middens distributed along different altitudes, relief, and mountain aspect. Since the subspecies geographic range is extremely restricted, these middens were enough to cover the local habitat variability. We visited each midden 6 times between the fall and winter of 2015 to measure variables of weather, relief, plant structure and estimate the volume of cones stored by the resident squirrels (response variable). During the visits, we also record the sex of the resident squirrels, by visual inspection using binoculars.

To estimate the volume of cones cached by each squirrel, we took advantage of the fact that the animals do not cache the cones loose in the scale pile but deposit them

into holes, here called “pits”, excavated in the scale pile or in the soil (Finley 1969). Thus, we used the number of pits, counted during the cache season, as proxy of the volume of cones cached in each midden. This method follows Gurnell (1984), but in the present study we did not excavate the pits to count the number of cones inside the pits, since it would disturb the midden of these endangered squirrels. The cache size estimates occurred between August 24 and September 12, an interval short enough to avoid any bias caused by the accumulation of cones along the cache season (LM, β = 0.03758, t = 1.726, p-value = 0.096). We also recorded the presence of dead logs near middens, because they are occasionally used to store cones (Finley 1969), but since the presence of logs had no detectable effects on the preliminary results, we excluded the variable from the analysis.

For the weather variables, we use a handheld weather meter (Kestrel 3000) to measure the air temperature (°C) and relative humidity (%), and a digital soil thermometer (HANNA HI45-30) to record soil temperature, the temperature inside of the scale pile, and inside of the pits (average value obtained from 3 randomly selected pits for each midden). Since we could measure only one midden at a time, the dial temperature variation, as well as the variation along different days, could easly mask any difference in middens microclimate. To make the measurements comparable, we used the data recorded by a meteorological station located at the top of the mountain (MGIO-Mt Graham Summit KAZSAFFO4) and by a data-logger (HOBO U23 Pro v2) buried in the ground as a baseline to allow comparison between middens. The weather station was used as baseline for the air temperature and humidity whereas the buried data-logger was used as baseline for the temperatures of soil, pits and scale piles. Since both baselines are fixed in space, by subtracting the baseline values from the values recorded in the middens, taken at the same time, the resultant value is the difference between the midden and the baseline,

which is a value comparable between middens. This way, the temperature and humidity variables are the mean of the values measured and corrected, for each midden, during the 6 visits.

Autumn is a critical period for the storage of cones because it is warmer and drier compared to the microclimate under the snow over winter, thus it is important to keep the cones in cool and humid conditions to avoid spoilage before the snow fall. Since the middens are usually cooler than the soil and air temperature during the fall (mostly due to evaporative heat loss) we calculated an additional variable called "pit cooling effect". This variable was calculated by subtracting the air temperature from the pit temperature, and represents how much cooler is the pit interior compared with the external environment.

Since some species are reported to cache more in areas with longer snow cover periods [2], and considering that memory and learning are important factors that modulate species behavioral [28], we decided to test the effect of snow cover duration on cache size. To do so, we capitalized on the fact that in 2015 all middens were covered by snow on December 12, and so we used the percentage of soil still covered by snow within a 5-m radius of each midden between 10 and 12 of March of 2016 as a proxy for the speed of snowmelt at each midden. We decided to use data from the 2015-2016 winter based on the premise that snow melting speed does not vary randomly along the mountain, but follows a pattern defined by relief and microclimatic factors [29]. Thus, a total of seven microclimatic variables were tested in the present work (Table 1; S2 Dataset).

Table 1. Explanatory variables.

Variables	Type	Unit
Altitude	Relief	Meters

Declivity	Relief	Degrees
Aspect*	Relief	Bearing
Soil temperature*	Microclimate	°C
Air temperature*	Microclimate	°C
Air humidity	Microclimate	°C
Pit temperature	Microclimate	°C
Scale pile temperature	Microclimate	°C
Pit cooling effect	Microclimate	°C
Snow melt speed	Microclimate	Percentage
Forest cover	Forest structure	Percentage
Tree density*	Forest structure	Ind/ha
Live tree density	Forest structure	Ind/ha
Dead tree density	Forest structure	Ind/ha
Live conifer density	Food availability	Ind/ha
Engelmann spruce	Food availability	Ind/ha
Douglas Fir	Food availability	Ind/ha
Ponderosa pine	Food availability	Ind/ha
Mushroom productivity	Food availability	Grams
Resident squirrel sex	Sexual	Binary

List of tested explanatory variables with the respective units. The principal explanatory variables, marked by a *, were selected by PCA analysis and were used to create models for subsequent model selection analysis.

To record the topographic relief variation, we used a GPS unit to record the altitude of each midden, a clinometer to record the declivity and a compass to record the aspect. For vegetation variables, between between 10 and 12 August, we used a densitometer to record the forest cover for each midden and recorded the arboreal community with diamater at breast height (DBH) ≥ 10 cm by using 4 transects of 30x5 meters, starting from the midden in the four cardinal directions. The density of live conifers in the transects was used as a proxy for the availability of cones near each midden. This proxy is based on the premise that the mean cone production of the conifer

species did not vary across the study area, which was tested and confirmed by the Moran's I test for spatial autocorrelation, using data from cone counting for 73 trees of the 3 species that produced cone crops in 2015 (Engelmann spruce: n= 33, p-value= 0.597; Douglas fir: n=35, p-value= 0.59; Ponderosa pine: n= 5, p-value= 0.269), nor was correlated with mountain aspect, which was tested and confirmed by linear models (Engelmann spruce: β = 0.694, t = 1.109, p = 0.276; Douglas fir: β = 0.001, t = 0.389, p = 0.7; Ponderosa pine: β = -0.008, t = -0.695, p = 0.537). From the tree transect data, we also calculated the trees density within the midden vicinity, density of live trees, density of dead trees, density of Englemann spruce, Douglas fir and Ponderosa pine, which are the main tree species that produced cones.

Between 3 and 7 August, we also estimate the productivity of mushrooms, an alternative food resource used and cached by squirrels [19,30], and their availability may interfere with the behavior of the animals. Mushroom productivity, recorded in grams, was estimated through 4 transects of 10x1 m, starting from the midden in the four cardinal directions. All mushrooms of all species known to be consumed by the squirrels [30,31] were collected and weighed.

To analyze the data, we verified the normality of the variables, using the Shapiro-Wilk test, and normalized the variables when needed. The response variable, number of pits, was log transformed to fulfill normality requirements, and the slope aspect, a circular variable, was tested for uniformity using a Kuiper uniformity test [32]. To allow the slope aspect variable to be analyzed together with the other linear variables, we linearized the variable by dividing it along the northeast-southwest axis (bearings 45° and 225°, where north is 0°\360°), and transfering the records in the northwest semicircle to the southwest semicircle. This way, the resultant semicircle can be treated as a linear variable, ranging from bearing 45° to 225°, where 45° represents a place where the soil slope is facing the

northeast, whereas 225° represents a place where the soil slope is facing towards southwest. We decided to use a northeast-southwest axis due visual and preliminary inspections of the raw data, which pointed to maximal variation along the northeast-southwest axis.

Due to the large number of explanatory variables, to avoid type I statistical errors, we divided the variables in biotic and abiotic and used Principal Component Analysis (PCA) to identify and delete the variables with high similarity within each group [33]. In this process, we used the PCA results for each group, selecting the more dissimilar variables based on the dissimilarity values within the first two dimensions, testing whether the variables were correlated with other similar variables, and then, deleting the correlated variable with smaller dissimilarity values. We performed a PCA again with the remaining biotic and abiotic variables, repeating the process to keep only the most dissimilar explanatory variables without correlations. After reducing the number of explanatory variables, we used a multiple competing hypothesis approach [34] to evaluate the effect of these remaining variables on the number of pits excavated by the squirrels.

For the competing hypothesis approach, we created a set of Generalized Linear Models (GLM), in which each model uses a different combination of one or two explanatory variables to explain the response variable (number of pits). For the model with two explanatory variables, we tested for both summatory and interaction effects between the explanatory variables, however, since interaction models performed poorly when compared to summation models, they were removed of the analisys. We did not test models with more than two variables due to the modest sample size of the dataset. A null model was created by using aleatory generated numbers as an explanatory variable, and the Akaike Information Criterion corrected to small sample size was used to compare the models [34]. For each model, we calculated the wAICc, a parameter of the relative

likelihood, and the ΔAICc, a parameter of relative difference between models. Models with ΔAICc < 2 were considered equally plausible. To reinforce the analysis, we calculated frequency πi, which is a bootstrap method to calculate the frequency in which a model *i* is selected as the best model in the set in 10000 random resamples of the dataset [34].

Finally, due to differences in reproductive strategy between males and females, the peak of reproductive energy expendure occurs in early spring for males and late spring for females [9]. Considering that in early spring, the males rely on cached food to maintain energy demanding reproductive activities, we tested the effect of sex of the resident squirrel on the cache size using Student's t-test and linear model regression. All statistics were performed using the R software [35] software, using the packages "circular" [36], "bbmle" [37], "FactoMineR" [38], "akima" [39], "plotrix" [40] and "ape" [41].

Results

From the 40 selected middens, 12 were excluded due to migration/death of the resident squirrel during the study. From the 28 remaining, 15 were occupied by males, 11 by females and 2 animals of unknown sex. The 28 remaining middens were evenly distributed in all aspects of the mountain slopes (Kuiper's Test of Uniformity, $p > 0.15$).

The PCA analysis reduced the 19 initial explanatory variables (excluding the resident squirrel sex, which is a binary variable) to only 4 principal explanatory variables, which are: air temperature, soil temperature, aspect and the density of trees in the midden vicinity (S1 Fig). The other 3 vegetation variables, 2 relief variables, 5 climate variables and 5 food availability variables were removed from the analysis due to their correlation with at least one of the four more dissimilar variables. Based on the four selected explanatory variables, eleven models were created, including the null model, and

compared using the Akaike Information Criterion method (Table 2). The model with highest explanatory power included the summed effects of relief aspect and density of trees as predictor variables (Fig 1), with the middens located in the northeastern slopes containing more pits than the middens in the southwestern slopes. At the same time, in the southeastern slopes, the middens surrounded by more trees contained fewer pits than the middens with less trees in its vicinity. This negative correlation between density of trees and number of pits was not observed in the northeast-facing slopes.

Table 2. Variables affecting red squirrel cache size.

Models	ΔAICc	Df	wAICc	πi
GLM10: NP ~ Aspect + Density of Trees	0.0	4	0.7161	0.599
GLM3: NP ~ Aspect	3.4	3	0.1337	0.061
GLM8: NP ~ Aspect+ Air Temperature	4.2	4	0.0865	0.220
GLM6: NP ~ Aspect+ Soil Temperature	5.3	4	0.0518	0.035
GLM5: NP ~ Soil Temperature + Air Temperature	10.3	4	0.0041	0.058
GLM4: NP ~ Density of Trees	11.5	3	0.0022	0.001
GLM9: NP ~ Air Temperature + Density of Trees	11.6	4	0.0021	0.017
GLM7: NP ~ Soil Temperature + Density of Trees	12.4	4	0.0015	0.003
GLM1: NP ~ Soil Temperature	12.9	3	0.0011	0.001
GLM2: NP ~ Air Temperature	14.5	3	<0.001	0.000
GLM0: NP ~ Null (aleatory generated values)	15.1	3	<0.001	0.000

Results of the model selection for Number of Pits (NP) excavated by Mt. Graham red squirrels. We fitted generalized linear models and calculated the corrected Akaike relative difference (ΔAICc), relative likelihood (wAICc) and a bootstrap model selection frequency (πi) to all models. Models with ΔAICc ≤ 2 were considered as equally plausible. The πi parameter was calculated based on 10.000 permutations.

Fig 1. Cache size in response to site aspect and tree density. Predicted number of pits excavated by Mt. Graham red squirrels in response to the site aspect (linearized in the x-axis), and density of trees (y-axis), based on the best fitted model. The black points represent the sampled middens.

The best model explained 44% of the observed variation in midden number of pits and received a wAICc = 0.7162, a frequency πi = 0.59, by far the most plausible model

(Table 2). Aspect was clearly the most important variable, since it was also present in the four models with higher explanatory power, while the tree density variable was, by itself, a weak variable and obtained only the sixth position in the rank of better models.

Due to its categorical nature, the resident squirrel sex was not included in the PCA and competing hypothesis analysis, instead, we divided the middens according to the sex of the resident squirrel and compared the two groups with student t-tests. We found differences in the number of pits excavated by males and females (t = 2.59, df = 23.78, p = 0.016), with males digging 47% more pits than females (Fig 2). We found marginal differences in the aspect of middens of males and females (t = -0.105, df = 20.196, p-value = 0.054), with more male squirrels being found on slopes with a northeastern aspect and more females in the southwestern facing areas (Fig 3). We found no evidence of an aspect effect on the cache behavior of males (β = -0.0006, t = -0.603, p = 0.556), whereas for females, the effect of the aspect was strong (β = -0.359, t = -7.877, p << 0.01), with females on northeastern slopes digging more pits than the females on the southwest facing areas (Fig 4).

Fig 2. Cache size of males and females. Differences in the number of pits excavated by male and female red squirrels on Mt. Graham, Arizona, USA.

Fig 3. Cache size along the mountain aspect. Circular distribution of the sampled middens along the mountain aspect. The radial axis represents the number of pits excavated by each resident squirrel. The distance between points in the picture does not represent geographical distances at the study site. The sex of the resident squirrels is indicated by blue points for males, red triangles for females and black diamonds for squirrels with unknown sex.

Fig 4. Differences in the response of males and females to site aspect. Regression of the number of pits excavated by males (blue points) and females (red triangles) in response of the aspect (linearized in the x-axis). For the description of the linearization process of the aspect variable, see the methods section.

Discussion

We found evidence to support our hypothesis about the existence of behavioral variation in the cache behavior of Mount Graham red squirrels, so that the number of pits excavated by these rodents changes according to the site aspect, density of trees and the sex of the resident squirrel. The data did not support the existence of temperature related behavioral variation, as squirrels displayed no response to any of the tested temperature variables. We found support for the existence of behavioral shifts in response to topographic relief, with middens in the northeastern slopes of the mountain containing more pits than in the southwestern slopes. This response was driven by females that strongly responded to aspect, whereas males did not. We found no support for behavioral shifts in response to food availability.

The absence of a response in the squirrel’s cache behavior to altitude, with the variable even excluded during the PCA, is not surprising considering that altitude per se (i.e. vertical distance between a point and sea level) did not have causative effects on biological systems. Instead, altitude must be considered as a strong proxy of other environmental variables, such as barometric pressure, temperature and humidity, which in turn have effects on biological systems [24]. Considering that temperature and humidity were also poor predictors of squirrel cache behavior, it is not a surprise that altitude was excluded by the PCA.

The absence of a response evidenced in the squirrel’s cache behavior to variation in the temperature along the mountain is surprising given how important temperature is to cone preservation [12,42] and thus to squirrel survival. However, several factors could interfere and/or preclude the emergence and maintenance of such behavioral responses. First, mean air temperature varied only 4.7 °C between middens, it is possible that squirrels simply lack the capability to detect such small variation and thus, do not respond

to these small temperature variations, independent of its importance to cone preservation. Even if squirrels are not capable of perceiving this variation in mean temperature, increases of just a degree or two are enough to impact cone preservation (J Koprowski, unpublished data), and a minimum increase of 1.5 °C is expected due to climate change before 2100 [22].

Another possible explanation is that the squirrels did not respond to the mean temperature, but to the occurrence and duration of extreme temperature events, such as observed in *Perisoreus canadensis* [13]. For food preservation, one or few events of extreme temperatures may cause cache spoilage through frost thaw or, in the case of red squirrels, by cone dehydration [12,13]. However, extreme temperature events were not detectable by the current experiment design, and therefore, a more specific experiment would be required to clarify this issue. This is a question with high conservation value, since it relates to whether the Mt. Graham red squirrels have behavioral ways to deal with the climate change predicted to the region [22].

The present study observed the effect of the site aspect, density of trees and sex of the resident squirrel on cache size, however, it was not possible to completely isolate and precisely estimate the effect of each one of these three variables on the animals' cache behavior. The isolation and evaluation of these three effects was hindered by the unexpected unequal distribution of males and females in the montain slopes, along the Notheast-Southwest axis. The loss of 30% of the middens due to squirrel mortality/migration during the sampling period further reduced the sample size and therefore limiting the statistical options available for data corrections. To precisely estimate the effect of aspect, density of trees and squirrel sex on cache size, a study with standardized sample size for males and females along the mountain aspect is

recommended, using an initial sample size big enough to compensate the low annual survival rate of Mt Graham red squirrels [20].

Althouth the exact mechanisms underlying the observed results are still not clear, there are some possible explanations that fit the observed data. A possible mechanism driving the effect of both aspect and density of trees on cache size is the incidence of solar radiation. In the northern-hemisphere, the southern mountain slopes receive sunlight at a more perpendicular angle than the northern slopes, and the forest canopy blocks more solar radiation from reaching the ground when the light came from a less perpendicular angle, such on the northern slopes [43]. Together, aspect and density of trees cause the northern slopes to be more shaded than southern slopes. This way, solar radiation might explain why cache size varies along the northeast-southwestern axis of the aspect, as observed for female squirrels, but its consequences on the squirrel cache are not clear.

Another possible mechanism underlying the observed differences in responses of males and females to tree density is related to the individual's perception of predation risk. Differences in boldness, vigilance and risk perception between sexes are a relatively common phenonema [44], and vigilance activities do interfere with foraging efficiency [45]. Since avian predation is a significant cause of mortality in the Mt. Graham squirrel population [10,20] and females have a higher annual survival rate than males [20], it is possible that tree density has different effects on risk perception for males and females, affecting their respective cache efficiency, effort and therefore cache size.

Our results document the existence of cache related behavioral variation in response to the topographic relief, density of trees and individual sex, in an endangered red squirrel subspecies. Although the mechanism by which these variables affect squirrel cache behavior are still not clear, it may be unrelated to site temperature, as would be usually expected based on the literature. Since temperature is important to cone

preservation [12], and the Mt. Graham red squirrels did not appear to behaviorally respond to temperature, the future increase in temperature may turn the Pinalenõ Mountains into unsuitable forest for these red squirrels. In the face of already unavoidable climate change, sky island complexes, such as the Madrean Archipelago [25,46], can be used as natural laboratories, providing us the opportunity to better understand how species characteristics, including behavior, affects its persistence capability. This may also allow us to develop and improve our conservation methods to protect populations on the edge of their distributions and on the edge of extinction.

Acknowledgements

We would like to thank to the Conservation Research Laboratory of the University of Arizona, Arizona Agriculture Experiment Station and the USDA Forest Service for logistic and financial support, to the Coordenação de Aperfeiçoamento de Pessoal de Nível Superior (CAPES) and Universidade Estadual Paulista "Júlio de Mesquita Filho" (UNESP-RC) for providing a one-year studentship at the study site, and to the Conselho Nacional de Desenvolvimento Científico e Tecnológico (CNPq) for a studentship in Brazil.

References

1. Sutton AO, Strickland D, Norris DR. Food storage in a changing world: implications of climate change for food-caching species. Clim Chang Responses. 2016;3: 12. doi:10.1186/s40665-016-0025-0
2. Wall SB Vander. Food hoarding in animals. University of Chicago Press; 1990.
3. Hadj-Chikh LZ, Steele MA, Smallwood PD. Caching decisions by grey squirrels:

a test of the handling time and perishability hypotheses. Anim Behav. 1996;52: 941–948. Available: http://psycnet.apa.org/psycinfo/1996-06811-009

4. Preston SD, Jacobs LF. Mechanisms of cache decision making in fox squirrels (*Sciurus niger*). J Mammal. 2009;90: 787–795. doi:10.1644/08-MAMM-A-254.1

5. Tamura N, Hashimoto Y, Hayashi F. Optimal distances for squirrels to transport and hoard walnuts. Anim Behav. 1999;58: 635–642.

6. Smith CC. The coevolution of pine squirrels (*Tamiasciurus*) and conifers. Ecol Monogr. 1970;40: 349–371. doi:10.2307/1942287

7. Cox PG, Rayfield EJ, Fagan MJ, Herrel A, Pataky TC, Jeffery N. Functional evolution of the feeding system in rodents. PLoS One. 2012;7: e36299. doi:10.1371/journal.pone.0036299

8. Hurley T a, Robertson RJ. Variation in the food hoarding behaviour of red squirrels. Behav Ecol Sociobiol. 1990;26(2): 91–97.

9. Archibald DW, Fletcher QE, Boutin S, McAdam AG, Speakman JR, Humphries MM. Sex-specific hoarding behavior in North American red squirrels (*Tamiasciurus hudsonicus*). J Mammal. 2013;94: 761–770. doi:10.1644/12-MAMM-A-213.1

10. Schauffert CA, Koprowski JL, Greer VL, Alanen MI, Hutton KA, Young PJ. Interactions between predators and Mt. Graham red squirrels (*Tamiasciurus hudsonicus grahamensis*). Southwest Nat. 2002;47: 498. doi:10.2307/3672515

11. Gendron RP, Reichman OJ. Food perishability and inventory management: a comparison of three caching strategies. Am Nat. 1995;145: 948–968. Available: http://www.jstor.org/stable/2463174

12. Finley Jr RB. Cone caches and middens of *Tamiasciurus* in the Rocky Mountain region. Misc Publ Museum Nat Hist Univ Kansas. 1969;51: 233–273. Available:

http://scholar.google.com/scholar?hl=en&btnG=Search&q=intitle:Cone+caches+and+middens+of+Tamiasciurus+in+the+rocky+mountain+region#0

13. Waite TA, Strickland D. Climate change and the demographic demise of a hoarding bird living on the edge. Proc R Soc B Biol Sci. 2006;273: 2809–2813. doi:10.1098/rspb.2006.3667

14. Robertson BA, Rehage JS, Sih A. Ecological novelty and the emergence of evolutionary traps. Trends Ecol Evol. 2013;28: 552–560. doi:10.1016/j.tree.2013.04.004

15. Gross K, Pasinelli G, Kunc HP. Behavioral plasticity allows short-term adjustment to a novel environment. Am Nat. 2010;176: 456–464.

16. Hampe A, Jump AS. Climate relicts: past, present, future. Annu Rev Ecol Evol Syst. 2011;42: 313–333. doi:10.1146/annurev-ecolsys-102710-145015

17. Lesica P, Allendorf FW. When are peripheral populations valuable for conservation? Conserv Biol. 2015;9: 753–760.

18. Hope AG, Malaney JL, Bell KC, Salazar-Miralles F, Chavez AS, Barber BR, et al. Revision of widespread red squirrels (genus: *Tamiasciurus*) highlights the complexity of speciation within North American forests. Mol Phylogenet Evol. 2016;100: 170–182. doi:10.1016/j.ympev.2016.04.014

19. Granillo KA, Froehlich GF. Mt. Graham red squirrel natural history and pre-observatory construction conservation efforts. In: Sanderson HR, Koprowski JL, editors. The last refuge of the Mt Graham red squirrel. The University of Arizona Press; 2009.

20. Goldstein EA, Merrick MJ, Koprowski JL. Functional semelparity drives population dynamics and endangers a peripheral population. Biol Conserv. 2017;205: 52–59. doi:10.1016/j.biocon.2016.11.017

21. Koprowski JL, Doumas SL, Merrick MJ, Oleson B, Posthumus EE, Jessen TG, et al. It's lonely at the top: biodiversity at risk to loss from climate change. Merging science and management in a rapidly changing world: Biodiversity and management of the Madrean Archipelago III and 7th Conference on Research and Resource Management in the Southwestern Deserts. Tucson, AZ, USA; 2013. pp. 53–59.

22. IPCC. Climate change 2014: Synthesis report. Contribution of working groups I, II and III to the Fifth assessment report of the Intergovernmental Panel on Climate Change. Geneva, Switzerland; 2014. doi:10.1017/CBO9781107415324

23. Wong BBM, Candolin U. Behavioral responses to changing environments. Behav Ecol. 2015;26: 665–673. doi:10.1093/beheco/aru183

24. Körner C. The use of "altitude" in ecological research. Trends Ecol Evol. 2007;22: 569–574. doi:10.1016/j.tree.2007.09.006

25. Warshall P. The Madrean Sky Island Archipelago: a planetary overview. In: DeBano LH, Ffolliott PH, Ortega-Rubio A, Edminster GJG, Hamre RH, B. C, editors. Biodiversity and management of the Madrean Archipelago: The sky islands of Southwestern United States and Northwestern Mexico. Tucson, AZ, USA: Fort Collins, CO: U.S. Department of Agriculture, Forest Service, Rocky Mountain Forest and Range Experiment Station; 1995. pp. 6–18.

26. Correa-Metrio A, Bush M, Lozano-García S, Sosa-Nájera S. Millennial-scale temperature change velocity in the continental northern neotropics. PLoS One. 2013;8: 1–11. doi:10.1371/journal.pone.0081958

27. Gurnell J. Home range, territoriality, caching behaviour and food supply of the red squirrel (*Tamiasciurus hudsonicus fremonti*) in a subalpine lodgepole pine forest. Anim Behav. 1984;32: 1119–1131. doi:10.1016/S0003-3472(84)80228-6

28. Mery F, Burns JG. Behavioural plasticity: an interaction between evolution and experience. Evol Ecol. 2010;24: 571–583. doi:10.1007/s10682-009-9336-y

29. Abudu S, Sheng Z ping, Cui C liang, Saydi M, Sabzi HZ, King JP. Integration of aspect and slope in snowmelt runoff modeling in a mountain watershed. Water Sci Eng. 2016;9: 265–273. doi:10.1016/j.wse.2016.07.002

30. Hendricks P, Hendricks LM. Use of conifers by red squirrels (*Tamiasciurus hudsonicus*) in Montana for drying and caching mushrooms. Northwest Nat. 2015;96: 240–242. doi:https://doi.org/10.1898/1051-1733-96.3.240

31. Koprowski JL, Greer VL, Merrick MJ. Mt. Graham red squirrel monitoring program - 2015 Annual report. Tucson, AZ, USA; 2015.

32. Pewsey A, Neuhäuser M, Ruxton GD. Circular statistics in R. Oxford University Press; 2013.

33. Jolliffe IT. Principal Component Analysis [Internet]. 2nd ed. New York: Springer-Verlag; 2002. doi:10.1007/b98835

34. Burnham KP, Anderson DR. Model selection and multi-model inference: a practical information-theoretic approach. 2nd ed. Springer; 2010.

35. R Development Core Team. R: A language and environment for statiscal computing [Internet]. Vienna, Austria: R Foundation for Statistical Computing; 2016. Available: http://www.r-project.org

36. Agostinelli C, Lund U. R package “circular”: circular statistics [Internet]. 2011. Available: https://r-forge.r-project.org/projects/circular/

37. Bolker B, R Development Core Team. bbmle: tools for general maximum likelihood estimation. R package version 1.0.20. 2017.

38. Le S, Josse J, Husson F. FactoMineR: an R package for multivariate analysis. J Stat Softw. 2008;25: 1–18. doi:10.18637/jss.v025.i01

39. Akima H, Gebhardt A. akima: interpolation of irregularly and regularly spaced data. R package version 0.6-2. 2016.

40. Lemon J. Plotrix: a package in the red light district of R. R-News. 2016. p. R-News, 6(4): 8-12.

41. Paradis E, Schliep K. ape 5.0: an environment for modern phylogenetics and evolutionary analyses in R. Bioinformatics; 2018.

42. Smith AA, Mannan RW. Distinguishing characteristics of Mount Graham red squirrel midden sites. J Wildl Manage. 1994;58: 437–445. doi:http://dx.doi.org/10.2307/3809314

43. Courbaud B, De Coligny F, Cordonnier T. Simulating radiation distribution in a heterogeneous Norway spruce forest on a slope. Agric For Meteorol. 2003;116: 1–18. doi:10.1016/S0168-1923(02)00254-X

44. Elgar M. Predator vigilance and group size in mammals and birds: a critical review of the empirical evidence. Biol Rev. 1989;64: 13–33. doi:10.1111/j.1469-185X.1989.tb00636.x

45. Verdolin JL. Meta-analysis of foraging and predation risk trade-offs in terrestrial systems. Behav Ecol Sociobiol. 2006;60: 457–464. doi:10.1007/s00265-006-0172-6

46. Parmesan C. Ecological and evolutionary responses to recent climate change. Annu Rev Ecol Evol Syst. 2006;37: 637–669. doi:10.1146/annurev.ecolsys.37.091305.110100

Supporting Information Captions

S1 Figure: Principal Component Analysis. PCA, showing the multivariate variation of the biotic (a) and abiotic (b) explanatory variables. Variables with similar variation were tested for correlation by the Pearson's correlation coefficient, when correlations were found, the most dissimilar variable was kept while the other was discarded. The resulting biotic and abiotic variables were put all together and the process was repeated (c). In the end pro the process, only the four variables (tree density, soil temperature, air temperature and aspect in a Northeast-southwestern axis) remained and therefore were used in the model selection.

S2 Dataset: Complete dataset. .CSV file containing the data used in the present study.

We thank the reviewers, for provide such a helpful feedback. Here are the responses to the questions.

Reviewer #1: This manuscript explores a highly fascinating behaviour 'caching variation in response to environmental and individual factors (gender)' in an endangered tree squirrel that inhabits a mountain range at the edge of the species distributional range. The latter is critical as these populations are subject to different evolutionary drivers than populations at the core of the distribution and therefore of particular interest when it comes to understanding behaviours and adaptions in a changing world.

The manuscript is exceedingly well written and a very interesting read. My only comment is that given the aim of the authors, this manuscript establishes a baseline and as a concluding thought I would encourage the research group to repeat this study in the future once climate change effects have progressed to see how this is affecting caching decisions and conclusions drawn to date. This would also show if squirrels respond to extreme temperature events. In this context a control study area at the core of the distribution not subject to extreme temperature fluctuations would be helpful for comparison.

Specific comments

1. Line 55: the statement about distance here lacks context. Is it really distance per se or more an increase in predation risk which makes retrieving this item for other individuals not familiar with the precise location of the valuable item more risky?

R: Indeed, the statement was unclear. We added some words to make the background clearer. But we didn’t add a complete explanation since it is provided in the cited manuscript.

Since squirrels usually cache food in the surroundings of the food source, farther places are safer in terms of conspecific pilferage. However, it takes more time and energy to travel farther before cache an item, increasing the general cost of the cache behavior by increasing the energy expended/energy retrieved (when the food is recovered) and reducing the energy cached/foraging time. This way, only more valuable food items worth the longer travel to a safer caching spot.

2. Line 57: From a European perspective it would perhaps be helpful here at first mention of this species (as opposed to Mt Graham red squirrel above) to at least once in addition to the scientific name to say the North American red or pine squirrel (Tamiasciurus hudsonicus) to avoid confusion for general readers.

R: Thank you for highlighting this issue. The words were added.

3. Line 64: should be 'depend'

R: We corrected the phrase grammar by changing the previous word (“caches” to “cache”).

4. Lines 68 and 71 - you have given Waite and Strickland 2006 here as full text when all other references are given as numbers - in your list it is at 42 and this should be changed.
R: The citations were corrected, making sure to meet the journal’s guidelines.

5. Line 111 - I wonder if these "absolute" predictions need to be modified or set into context? I assume it is a given that predictions 1 and 2 (lines 107-110) are also subject to local cone (mushroom) availability per se (your variable of density of trees in midden vicinity?
R: To avoid make unnecessarily complex predictions, we carefully created only three simple and direct predictions, each one with single predictor subject (1. Large scale temperature, 2. microclimate, 3. food availability). Yes, we indeed expected these predictions to interfere with each other, and this is the reason why we make sure to use non-excluding predictions (the three can be truth and have summed effects). We even tested these interactions in the methods to some degree, always being careful to choose a less demanding statistic approach due to the limited sample size.

6. Line 196 'varied' should be 'vary'
R: The word was changed.

7. Lines 196 to 199 - as aspect is a critical factor in your later findings, did you include aspect as a variable here - did/would the sample of 73 trees allow this? One underlying assumption in your study is that all trees within midden vicinity are equal in terms of food availability and their density serves as a proxy. One might in principle expect aspect to matter in terms of cone production and this should be checked.
R: Thank you for highlight this issue. We added the information about the possible correlation between cone production and mountain aspect. The cones production was also not correlated with aspect.

Reviewer #2: This paper describes an important evolutionary adaptation: behavioural variability in the amount of food cached by the endangered Mount

Graham red squirrel (Tamiasciurus fremonti grahamensis). Authors tested the variation in cache size in response to microclimate variation, based on the following parameters: relief, vegetation (tree stand density), food availability and squirrel sex. Based on assumptions and predictions obtained from previous studies scientists expected that squirrels should locate bigger caches in areas:

1. Where the conditions to cone preservation are better and spoilage is expected to be reduced (colder areas).
2. With colder microclimates: in higher elevations and in slopes facing the northeast.
3. With higher cone availability.

To verify these hypothesis authors selected 40 middens occupied by squirrels distributed along different altitudes, relief, and mountain aspect. They visited each selected midden six times between the fall and winter of 2015.

Results of the study show, that much more pits were excavated in the northeast facing slopes. Surprisingly, the density of trees negatively affects the cache volume on southwest slopes only. The sex of the squirrel also affects the number of pits in the squirrel midden, males excavating more pits than females. Additionally, both sexes presented different responses to the mountain slope aspect (females excavating more pits on northeastern slopes, whereas male cache size did not vary with the slope aspect). Contrary to authors' predictions the squirrel's caching behaviour did not vary in response to midden microclimate variation.

However, although both the idea and conducted study are very interesting and worth publication, there are some aspects which require further explanation.

First of all, in my opinion (and as the authors mentioned in the discussion), the sample size may be not big enough for such multifactorial analysis. The second question is a type of distribution of studied middens – does it cover all habitat variability?

R: Indeed, due to the unexpected high mortality of squirrels during the winter, we worked always with the limitations of a small sample size in mind, choosing the less demanding statistical approaches, such reducing the number of variables before compare the models and never creating models with more than independent 2 variables. Despite our efforts, there is a limit to what is possible to do, and this is the reason why, in good faith, we added the warning in the discursion section (as mentioned by the reviewer).

About the distribution of the study middens, since the squirrel distribution is very limited, the sampling did cover most the habitat variability. We added a phrase in the manuscript about the subject. The middens coordinates are also provided in the supplementary material.

For me, the relationship between altitude, microclimate and the number of pits in the squirrel's midden is the most important and missing information which may

help us understand behavioural adaptation to the climate change. Authors should expect differences in caching squirrels' behaviour in regard to differences in elevation gradient. In my opinion this aspect should be deeply analysed and discussed. Maybe squirrels territories were located at the same elevation – it should be disclosed.

R: The altitude of the middens varied between 2770 and 3270 meters (as mentioned in the "study area" subsection), which is almost the entire range of altitude of the population. However, Altitude was not "deeply analyzed", because it was not a good predictor of squirrel cache size and was dropped in the variable reduction part of the analysis.

We added an entire paragraph to better explain the issue in the manuscript.

I am also wondering why authors analyzed differences between squirrels' sexes? Did they expect any sex-related differences in catching behaviour and if so, there should be an explanation.
It would be good to introduce potential readers for studied species biology and describe briefly the subject of the study, including sexual differences, body mass and social system.

R: We added more details about how the squirrel sexual differences and why it is expected to affect the animal dependence on cached food.

When you try to consider what causes could lead to the obtained results – the lack of behavioural reaction on micro-climate variability – the good way is to analyze the used methodology. It is possible that daily air temperature, which was measured in the meteorological station differs slightly from the daily air temperature in other places, like middens. The same could be applied to soil temperature monitored by data-logger between one (the same) place and other places (e.g. middens).
Other explanation which the author discussed in the manuscript is that the most important for catching behaviour was the maximum, not the average air temperature.

R: Indeed, we agree with the reviewer, and it was the used approach. But we did not discuss about possible differences between the measurements between the meteorological station and the middens, since this would result in constant error along the middens, and therefore, it would be automatically corrected by the used methods. As the reviewer mentioned, we preferred to discuss about possible extreme events (such as temperature peaks), which would require a very different sampling method, and therefore, are beyond the original design of the present study.

Abstract

P2 L22 - …" in response to climate"… - should be "in response microclimate"

R: the phrase was changed to "in response to microclimate"

Materials and Methods

P6 L131 – 134

"We visited each midden 6 times between the fall and winter of 2015 to measure variables of weather, relief, plant structure and estimate the volume of cones stored by
the resident squirrels (response variable). During the visits, we also record the sex of the
resident squirrels."

Could you please explain how did you record the sex of the resident squirrels? It's unclear – Did you measure all of these variables during each of the six visits? If not please add the information when did you measure each variable. As I understood the temperature in all middens was measured on the same day. How did you choose three pits from one midden for temperature monitoring? How accurate was your equipment for the weather variables (handheld weather meter Kestrel 3000) and a digital soil thermometer (HANNA HI45-30), it's possible that low accuracy can mask differences in temperature and humidity.

R: The sex of the resident squirrels was recorded by direct observation (using a binocular). It is a very easy and reliable method for sexing squirrels since they are usually up in the branches. We added the information in the manuscript.

We also add the information about when each variable was collected.

We didn't think the equipment precision was a problem, since it is a very reliable equipment. The technical specifications can be accessed in the respective equipment websites (±0,27°C for the HANNA HI45-30 and ± 0.04C for the Kestrel 3000).

Discussion

In this part of the manuscript, there is no explanation of why sex should generate differences in squirrel's catching behaviour.

R: The information was added in the methods section, as the reason of why we recorded squirrel sex.

Additionally, the authors' hypothesis about different levels in predation risk

between places with high and low tree steam density as an explanation in differences in squirrels' catching behaviour seems to be unconvincing. First of all, it is hard to believe that predation risk can vary between different mountain slope aspects.

R: We thank the reviewer to point that the argument was not clear. We make some changes to make it clearer. Indeed, the argument is not intended to explain the different responses of males and females to the mountain aspect, but only in relation to tree density.
